# Electrochemical Collisions of Individual Graphene Oxide Sheets: An Analytical and Fundamental Study

**DOI:** 10.1002/celc.201901606

**Published:** 2019-12-17

**Authors:** Christophe Renault, Serge G. Lemay

**Affiliations:** ^1^ MESA+ Institute for Nanotechnology University of Twente P.O. Box 127 7500 AE Enschede The Netherlands; ^2^ Physique de la Matière Condensée Ecole Polytechnique, CNRS, IP Paris 91128 Palaiseau France

**Keywords:** graphene oxide, impact electrochemistry, single-entity electrochemistry, migration, size characterization

## Abstract

We propose an analytical method based on electrochemical collisions to detect individual graphene oxide (GO) sheets in an aqueous suspension. The collision rate is found to exhibit a complex dependence on redox mediator and supporting electrolyte concentrations. The analysis of multiple collision events in conjunction with numerical simulations allows quantitative information to be extracted, such as the molar concentration of GO sheets in suspension and an estimate of the size of individual sheets. We also evidence by numerical simulation the existence of edge effects on a 2D blocking object.

## Graphical abstract

**Collision course**: The electrochemical signature from individual graphene oxide sheets colliding on an ultra‐microelectrode is analyzed quantitatively as a function of particle size, charge, and salt concentration.

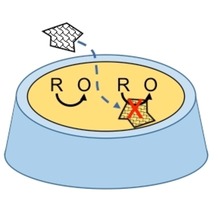

Graphene oxide (GO) is a two‐dimensional material, atomically thin but with lateral dimensions up to several hundred microns. The electronic, mechanical and chemical properties of this material have attracted considerable interest, not least for the fabrication of sensors.^1^ Traditional ways of preparing GO suspensions such as Hummer's method can produce material with high polydispersity in size, degree of oxidation, number of defects and, hence, conduction.^2^ This disparity leads to difficulties in obtaining consistent devices or understanding the properties of GO‐based materials. Techniques to simultaneously probe *in‐situ* several physicochemical properties of a GO suspension are thus desirable. Yang and coworkers measured by electrochemistry the iron content of individual reduced GO sheets modified with microperoxidase‐11 and hence deduced their degree of functionalization.^3^ More recently, Compton and coworkers used nano‐impact electrochemistry to probe the electro‐catalytic activity of single GO sheets decorated with Pd nanoparticles. Hence, they determined that only a fraction of the Pd nanoparticles are active upon collision of a GO on a carbon microelectrode.^4^ Here, we explore the analytical capabilities and limits of a different electrochemical detection scheme based on current blocking.^5^ The principle is illustrated in Figure 1A. A redox reporter (O/R) initially dissolved in solution is oxidized/reduced at an ultra‐microelectrode (UME) to produce a steady‐state faradaic current. When an insulating object masks a portion of the UME and blocks the electrochemical reaction, the steady‐state current decreases by a small fraction. In this communication we used the blocking strategy to detect insulating GO sheets.

**Figure 1:**
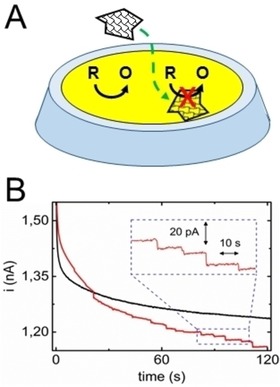
A) Principle of the electrochemical blocking method. The letters R and O correspond to the species FcMeOH and FcMeOH^+^, respectively. B) *i‐t* curves recorded with a 10 μm diameter gold UME in a 1 mM FcMeOH, 100 μM KNO_3_ aqueous solution in the absence (black curve) and presence (red curve) of 0.2 μg/mL of GO. The UME is biased at 0.3 V vs Ag/AgCl.

Our blocking experiments are carried out as follows. First, a 10 μm diameter gold UME is dipped in 1 mM ferrocene methanol (FcMeOH) solution and biased at 0.3 V vs Ag/AgCl wire to achieve a quasi‐steady‐state current (here the current is not perfectly constant), as shown by the black *i‐t* curve in Figure 1B. Subsequently, GO sheets with an average diameter of 530±370 nm, as measured by AFM (see SI, Figure S1), are introduced in solution. The GO stock solution is sonicated at least 30 min prior to the injection and GO concentrations≤1 μg/mL are used to ensure that only single‐layered sheets are present. Finally, a second *i‐t* curve, this time in the presence of GO, is recorded (red trace in Figure 1B). In this recording several decreasing current steps are observed. The inset Figure 1B shows a zoom on some of the current steps. The steps are sharp (rise time ≈20 ms, limited by our electronics) and present different sizes as well as different time intervals between steps. The appearance of steps indicates that individual GO sheets irreversibly adsorb on the UME surface and lay flat so that the oxidation of FcMeOH is blocked over a significant surface area. From this observation we can draw, at the single‐sheet level, two conclusions. First, individual GO sheets are insulating enough to block the electron transfer through the plane of the sheet. This observation is in agreement with the high electrical resistivity of GO (>10^3^ Ω⋅cm), and the thickness of a single GO sheet (≈1 nm) that should considerably slow down tunneling currents.^6^ Second, the GO sheets do not possess enough structural defects (e. g. broken C−C bonds) to let a significant amount of FcMeOH molecules (hydrodynamic radius 0.27 nm)^7^ pass through their structure. This last point is important since traditional conductivity measurements probe only the electronic conductivity but not the permeability, an important criterion for devices operating in wet environments.

A quantitative analysis of the current steps was performed as follows. First, we analyzed the rate of arrival of the GO sheets to the electrode. The rate of occurrence of the current steps, or frequency of GO collision on the UME (taken as the inverse of the average time interval between steps), was measured for different concentrations of GO and is plotted in Figure 2A. A linear relationship between the frequency of collision and the concentration of GO sheets is observed (red curve in Figure 2A; linear fit, *R* ^2^=0.996). This observation is in agreement with the two relevant modes of mass transport, diffusion and migration, which are both linearly dependent on the concentration of GO sheets. Migration also depends on the charge of a GO sheet and on the electric field in solution. The latter is determined by the potential at the electrode as well as the concentration of charged species such as K^+^, NO_3_^−^ and FcMeOH^+^. In order to separate the contributions of diffusion and migration, we increased the concentration of the electrolyte, KNO_3_, so as to progressively suppress the electric field.

**Figure 2:**
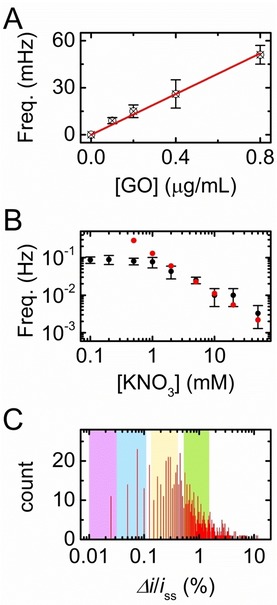
A) Collision frequency as a function of the mass concentration of GO. The error bars correspond to the standard deviation measured between five independent measurements. The red line is a linear fit *y*=*ax* with *a*=65+/−2 mHz/(μg/mL) and R^2^=0.996. The experimental parameters are: *E*_WE_=0.3 V; [FcMeOH]=0.9 mM; [KNO_3_]=10 mM; 163 collisions were recorded in total. B) Collision frequency as a function of the salt concentration. [GO]=0.2 μg/mL and [FcMeOH]=0.9 mM; the error bars correspond to the standard deviation measured on three independent experiments. The red dots were obtained by solving numerically the PNP equation. See text and Supporting Information for further details. C) Histogram of the relative step size; 723 steps are counted in total; bin size=0.025 %. The pink, blue, yellow and green rectangles correspond to the simulated range of step size produced by insulating disks of 250, 500, 1000 and 2000 nm diameter, respectively. See Supporting Information for further details.

The experimental frequency of collision as a function of the salt concentration is plotted with black dots in Figure 2B (log‐log scale). When increasing the concentration of KNO_3_ from 2 mM to 50 mM, the frequency of collision decreases by almost two orders of magnitude down to 0.003 Hz. This trend is consistent with the generation of FcMeOH^+^ at the electrode and the corresponding migration of anions toward the electrode. The relative contribution of the negatively charged GO sheets (zeta‐potential=‐40±10 mV, measured with a Zetasizer Nano ZS, Malvern) to current decreases upon adding supporting electrolyte. Previous studies realized on polystyrene microspheres and nanoparticles evidenced similar migration effects.5a, 5e Further increase of the salt concentration renders the GO dispersion unstable and thus prevents the observation of a purely diffusive regime where the frequency of collision is independent of the salt concentration.

For KNO_3_ concentrations lower than 2 mM, the frequency of collision is independent of the salt concentration with a value of 0.09 Hz. A reason for this crossover is suggested by noting that the concentration of supporting anions near a hemispherical electrode, $c_{-}\left(r\right)$ , is approximately given by the expression [Eq. (1)]:^8^

(1) $$c_{-}\left(r\right)=c_{salt}\left(1+\frac{c_{Red}^{0}}{2c_{salt}}\frac{a}{r}\right)$$

Here $c_{salt}$ is the bulk salt concentration, $c_{Red}^{0}$ is the bulk concentration of reduced ferrocene methanol, a is the radius of the electrode and r is the radial distance from the electrode (see SI for details). Accumulation of anions takes place near the electrode when$\;c_{salt}\;\leq c_{Red}^{0}/2$ , which coincides well with the observed crossover. This surplus anionic charge compensates the cationic oxidized species generated at the electrode. GO sheets approaching the electrode experience a strong ionic strength gradient, which can negatively influence their electrophoretic mobility. Simultaneously, the electric field, ℰ(r ), is modified compared to its value extrapolated from high supporting electrolyte ratios, ${ℰ}_{supp}\left(r\right)$ [Eq. (2)]:

(2) $$ℰ\left(r\right)=\frac{{ℰ}_{supp}\left(r\right)}{1+\frac{c_{Red}^{0}}{2c_{salt}}\frac{a}{r}}$$

That is, the electric field is suppressed near the electrode when$\;c_{salt}\;\leq c_{Red}^{0}/2$ , further hindering local migration. While a complete description of the electrophoresis of GO in concentration gradients is beyond our present scope, it appears plausible that the observed plateau behavior originates from concentration polarization at the electrode.

The instability of the GO suspension at high salt concentration means that there is no simple experimental way to access a purely diffusive regime. Determining the concentration of GO from the frequency of collision therefore requires an estimate of the contribution of migration. This can be done by solving the Poisson‐Nernst‐Planck (PNP) equation.^9^ Finite‐element methods (COMSOL Multiphysics) were used for this purpose and calculate a theoretical frequency of collision at different concentrations of KNO_3_ (see SI for details of the calculation). In our simulation the values of the diffusion coefficient (5×10^−13^ m^2^/s) and mobility (2.8×10^−8^ m^2^/V/s) of GO were determined independently by Dynamic Light Scattering (DLS, Zetasizer Nano ZS, Malvern) and directly substituted into our model, leaving the molar concentration of GO sheets as the only adjustable parameter. The theoretical frequency values are plotted in red in Figure 2B. They show excellent agreement between simulation and experiment in the intermediate salt concentration range (2 to 50 mM), where the frequency continuously decreases as the salt concentration increases. From this one‐parameter fit we extract that a mass concentration of 1 μg/mL of GO corresponds to a molar concentration of 150 fM of sheets, which corresponds to an average diameter of 530 nm. Interestingly, the data of Figure 2 indicate that at low salt concentration we are able to detect down to 15 fM of GO sheets in a few minutes. In comparison, a quantitative technique such as UV‐Visible absorption spectroscopy is limited to about 150 fM (0.003 OD at 450 nm in a 1 cm long cell containing 1 μg/mL of GO). The excellent sensitivity of the electrochemical collision method comes from its ability to detect individual GO sheets.

To ascertain the smallest sheet size detectable, we now turn to the size of the current steps. A histogram of the step size (normalized by the steady‐state current right before the step) is shown Figure 2C. A large distribution of step size (log scale) is evidence with a maximum at about 0.2 %. We performed numerical simulations to estimate the step size caused by disk‐shaped idealized GO sheets of four different sizes: 250, 500, 1000 and 2000 nm diameter. As Fosdick and al. previously evidenced, for a same object the step size can differ by a factor of roughly 4 depending if it falls at the center of the UME (where the flux is the lowest) or on the edge of the UME (where the flux is roughly 4 times higher than at the center).^10^ Consequently, we performed simulations of blocking disks positioned at the center and the edge of the UME. The minimum and maximum current steps caused by a 250, 500, 1000 and 2000 nm diameter sheet are indicated with, respectively, the pink, blue yellow and green shaded regions in Figure 2C. A large distribution of diameters (from 0.25 to 2 μm) is evidenced, in agreement with AFM measurements (Figure S1 in SI). The maximum of the step distribution (≈0.25 %) corresponds to a sheet size of about 1 μm in diameter while the AFM measurement shows an average diameter of 530±370 nm. This difference evidences that the shape of the step histogram does not provide directly the distribution of sheet size. The count of steps is biased by both mass transfer (small objects diffuse faster than larger ones and thus hit more frequently the electrode) and the resolution in step size (the smallest objects are not always counted). The limit of detection of our method is fixed by the resolution of the electrochemical measurement, which is about 0.2 pA (≈0.02 % step size). According to our simulations this step size corresponds to a sheet diameter of roughly 250 nm. In order to detect even smaller GO sheets, larger concentrations of redox reporter or smaller electrodes could be used.^11^ The width of the size distribution can also be accurately measured by using hemispherical UMEs instead of disk‐shaped UMEs.^12^

The collision of GO sheets raises the interesting question whether a 2D object is better at blocking than a 3D object with a similar cross‐section and how these two objects block the current. To tackle these questions, we performed additional numerical simulations. The diffusion profiles of FcMeOH oxidized on a UME (radius *a*) blocked with a sheet and a sphere are shown in the top and bottom panels of Figure 3A, respectively. The sheet and the sphere (represented in white in Figure 3A) have a similar length/diameter (0.2*a*) and position on the UMEs (centered at 0.7*a,* represented in grey in Figure 3A). Further details about the simulation are provided in SI. The magnitude of the relative step size produced by the disk and the sphere are 0.87 % and 3.77 %, respectively. These values are obtained by integrating the flux at the electrode in a revolved geometry (i. e. the disk and the sphere become an annulus and a toroid, respectively). Interestingly the sphere blocks about four times more current than the disk. To understand why a 3D object blocks more current than its 2D projection one must take a look at the concentration profiles. Indeed, the concentration profiles are significantly different depending on the object dimension. While the concentration of FcMeOH between the UME and the bead is constant and relatively low (blue color), a high concentration of FcMeOH (yellow color) is evidenced just at the surface of the sheet center with a strong gradient of concentration near the edge of the sheet. This difference of concentration gradient is emphasized in Figure 3B where the flux of FcMeOH at the electrode surface is plotted as a function of the radial position on the UME. The blue, red and green traces correspond to the flux at a bare UME, a UME blocked by a sheet and a UME blocked by a sphere, respectively. For the bare electrode we observe the classic “edge effect” or increase of flux at the edge caused by radial diffusion. In the presence of a sheet, the flux is null at the location of the object. However, the flux on the edges of the sheet is significantly increased compared to a bare UME. This means that some of the FcMeOH not consumed near the center of the sheet is instead able to diffuse to its edge. On the other hand the sphere does not cover the surface of the UME and thus the flux is not null under the sphere (except for the narrow region of the sphere in contact with the surface). Only a slight increase of flux is observed at the edges of the sphere and the flux decreases continuously upon approaching the point of contact with the surface.

**Figure 3:**
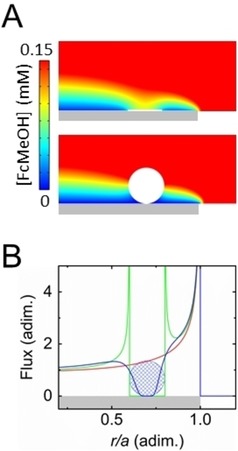
A) Simulated concentrations of FcMeOH (*C*_bulk_=1 mM) oxidized on a UME at a mass transfer limited regime. The top and bottom concentration sections correspond to a UME blocked with a sheet and a sphere respectively. The grey rectangle represents the electrode. The white color corresponds to the blocking object. The color scale was adjusted to emphasize the concentration gradient near the electrode and does not cover all the range of concentration. B) Flux profile of FcMeOH at the surface of the electrode as a function of radial position, *r*. The flux and the radial position are normalized with respect to the flux at the center of the UME (450 μmol/m^2^/s) and the radius of the electrode (*a*=1 μm), respectively. The red, green and blue traces correspond to a bare electrode, an electrode blocked with a sheet and an electrode blocked with a sphere, respectively. The blue grid‐patterned circle represents the blocking sphere.

The difference in flux profiles illustrates well the two different modes of blocking between a sphere and a sheet. While the sheet diminishes current by blocking electron transfer locally at the surface, the sphere hinders mass transport by rendering the volume above the electrode inaccessible. This highlights the fact that an object does not have to be in intimate contact with the electrode surface to produce an efficient blocking step. It also means that the step size encodes some information about the height of the object. This information is however convoluted with the shape and projected area of the object as well as its position on the electrode surface.

In conclusion, we successfully employed the blocking method to electrochemically detect single GO sheets as small as 250 nm and determine rapidly (c.a. 30 min) the molar concentration of extremely dilute suspensions of GO (few tens of fM). We provide a comprehensive analysis of the electrochemical signal with the support of numerical simulations.

## Experimental Section

Details on chemicals, electrochemical measurements, AFM, derivation of Equations (1) and (2), and numerical simulations are provided in the Supporting Information.

## Conflict of interest

The authors declare no conflict of interest.

## Supporting information

As a service to our authors and readers, this journal provides supporting information supplied by the authors. Such materials are peer reviewed and may be re‐organized for online delivery, but are not copy‐edited or typeset. Technical support issues arising from supporting information (other than missing files) should be addressed to the authors.

Supplementary

## References

[1] D. Chen , H. Feng , J. Li , Chem. Rev. 2012, 112, 6027–6053. 22889102 10.1021/cr300115g

[2] D. R. Dreyer , S. Park , C. W. Bielawski , R. S. Ruoff , Chem. Soc. Rev. 2010, 39, 228–240. 20023850 10.1039/b917103g

[3] D. Li , J. Liu , C. J. Barrow , W. Yang , Chem. Commun. 2014, 50, 8197–8200. 10.1039/c4cc03384a 24927153

[4] R. Miao , L. Chen , L. Shao , B. Zhang , R. G. Compton , Angew. Chem. 2019, 131, 12679–12682.

[5a] B. M. Quinn , P. G. van′t Hof , S. G. Lemay , J. Am. Chem. Soc. 2004, 126, 8360–8361; 15237976 10.1021/ja0478577

[5b] B.-K. Kim , A. Boika , J. Kim , J. E. Dick , A. J. Bard , J. Am. Chem. Soc. 2014, 136, 4849–4852; 24641496 10.1021/ja500713w

[5c] E. Lebègue , C. M. Anderson , J. E. Dick , L. J. Webb , A. J. Bard , Langmuir 2015, 31, 11734–11739; 26474107 10.1021/acs.langmuir.5b03123

[5d] J. E. Dick , A. T. Hilterbrand , A. Boika , J. W. Upton , A. J. Bard , Proc. Mont. Acad. Sci. 2015, 112, 5303–5308; 10.1073/pnas.1504294112 PMC4418878 25870261

[5e] A. Boika , S. N. Thorgaard , A. J. Bard , J. Phys. Chem. B 2013, 117, 4371–4380; 23092206 10.1021/jp306934g

[5f] J. Bonezzi , A. Boika , Electrochim. Acta 2017, 236, 252–259.

[6a] I. Jung , D. A. Dikin , R. D. Piner , R. S. Ruoff , Nano Lett. 2008, 8, 4283–4287; 19367929 10.1021/nl8019938

[6b] C. Miller , P. Cuendet , M. Graetzel , J. Phys. Chem. 1991, 95, 877–886.

[7] M. P. Longinotti , H. R. Corti , Electrochem. Commun. 2007, 9, 1444–1450.

[8a] K. B. Oldham , J. Electroanal. Chem. Interfacial Electrochem. 1988, 250, 1–21; 10.1016/0302-4598(87)85005-5 PMC4257492 25484449

[8b] C. Amatore , M. R. Deakin , R. M. Wightman , J. Electroanal. Chem. Interfacial Electrochem. 1987, 225, 49–63.

[9] J. Xiong , Q. Chen , M. A. Edwards , H. S. White , ACS Nano 2015, 9, 8520–8529. 26190513 10.1021/acsnano.5b03522

[10] S. E. Fosdick , M. J. Anderson , E. G. Nettleton , R. M. Crooks , J. Am. Chem. Soc. 2013, 135, 5994–5997. 23590646 10.1021/ja401864k

[11] J. E. Dick , C. Renault , A. J. Bard , J. Am. Chem. Soc. 2015, 137, 8376–8379. 26108405 10.1021/jacs.5b04545

[12] Z. Deng , R. Elattar , F. Maroun , C. Renault , Anal. Chem. 2018, 90, 12923–12929. 30284818 10.1021/acs.analchem.8b03550

