# Supplementary material for: Electrochemical Collisions of Individual Graphene Oxide Sheets: An Analytical and Fundamental Study
Source: ChemElectroChem. 2019 Dec 17;7(1):69–73. doi: 10.1002/celc.201901606 (PMC6973065; doi:10.1002/celc.201901606)
Supplement: Supplementary file 1 — Supplementary [file CELC-7-69-s001.pdf]

CHEMELECTROCHEM

Supporting Information

© Copyright Wiley-VCH Verlag GmbH & Co. KGaA, 69451 Weinheim, 2019

Electrochemical Collisions of Individual Graphene Oxide Sheets: An Analytical and Fundamental Study

Christophe Renault* and Serge G. Lemay*© 2019 The Authors. Published by Wiley-VCH Verlag GmbH & Co. KGaA. This is an open access article under the terms of the Creative Commons Attribution License, which permits use, distribution and reproduction in any medium, provided the original work is properly cited.An invited contribution to the Richard M. Crooks Festschrift

Table of contents

Chemicals and procedures -- p2

Electrochemical measurements -- p2

AFM measurements -- p3

Discussion on the collision frequency plateau at law salt concentration ------------------------------ p4

Finite-element Simulations --- p8

Chemicals and procedures

Graphene oxide (GO) in water suspension (4 mg/mL) was purchased from Graphenea Inc. (Cambridge, USA). Ferrocene methanol (FcMeOH), ferrocene dimethanol (FcDM), potassium nitrate, 96% ethanol, 98% sulfuric acid and 30% hydrogen peroxide were purchased from Sigma-Aldrich (Saint Louis, USA). All chemicals were used without further purification. DI-water (18.2 MΩ.cm, 2-4 ppb total organic content) was produced with a Milli-Q Advantage A10 (Millipore) purification system. PTFE Syringe filters with a pore size 0.1 µm were purchased from Merck Millipore.

The solutions of FcMeOH and FcDM were filtered with a 0.1 µm syringe filter. The glassware was cleaned with piranha solution (25%v H_2O_2, 75%v H_2SO_4) before every experiment. The concentrations of the FcMeOH and FcDM solutions were determined before the experiment using a 1 mm diameter glassy carbon electrode (BASi, West Lafayette, USA) and recording cyclic voltammograms (CVs) at different scan rates.

Electrochemical measurements

The measurements were performed on a vibration isolation table placed inside a Faraday cage. The current was recorded with a DDPCA-300 transimpedance amplifier from Femto (Berlin, Germany). A two-electrode configuration was used for the collision measurements. The working electrode was a 10 µm diameter gold UME successively polished with 1, 0.3 and 0.05 µm alumina slurry (5 min each) and then sonicated in water, ethanol and gently wiped on a cloth. The cleanliness of the UME was checked by optical microscopy and electrochemistry (CV of FcMeOH). The counter/reference electrode is a Ag/AgCl wire. Before each experiment, a CV of the redox probe was recorded to determine the potential needed to reach the mass transfer limit and compensate for any eventual drift of the Ag/AgCl reference wire. Typically, an overpotential of 0.3 - 0.5 V was used for chronoamperometric experiments. After addition of GO solution to the cell, the latter was gently shaken to homogenize the solution.

Determination of the sheet size by AFM

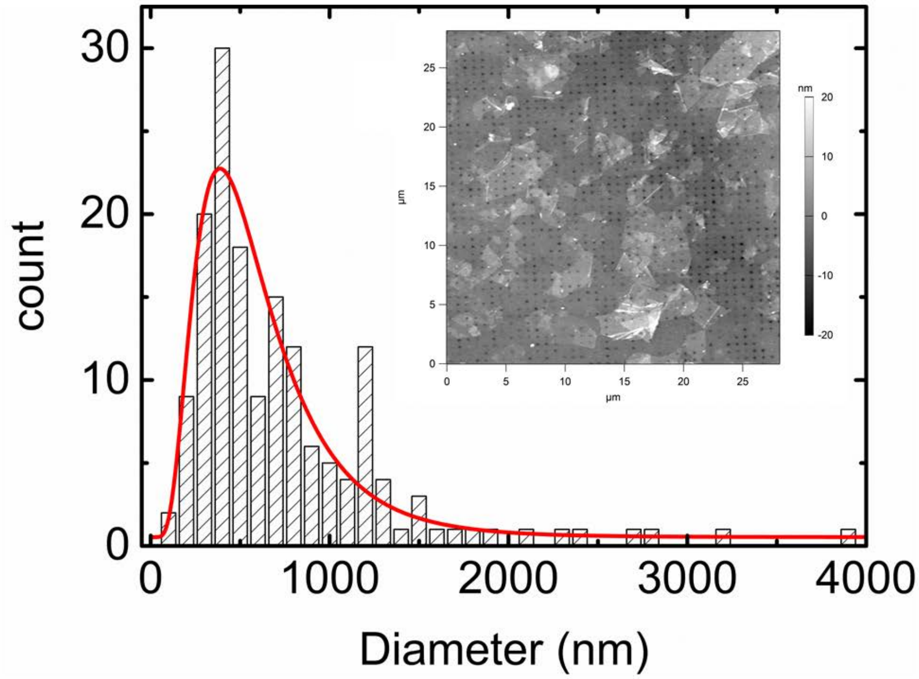

Figure S1. Sheet size was measured by AFM imaging on n_{tot} = 161 sheets. The red line is a fit of the experimental data to a Log-Normal distribution (median = 530 nm, mean 615 nm, standard deviation = 370 nm, R^2 = 0.863). The diameter was taken as the average of the longest and shortest axis passing through the centre of gravity of a sheet.

Atomic force microscopy (AFM) was carried out with an Asylum Cypher (Santa Barbara, CA) instrument. The measurements were performed in contact mode. GO sheets were deposited on a flat SiN_3 substrate (with small lithographied cavities, see regular black hole pattern on a typical AFM image in inset Figure S1). The substrate was incubated (1-2 h) in a 1 µg/mL GO in water suspension (sonicated 30 min beforehand) followed with a gentle rinsing step with DI water and terminated with a drying step at room temperature.

Discussion on the collision frequency plateau at law salt concentration

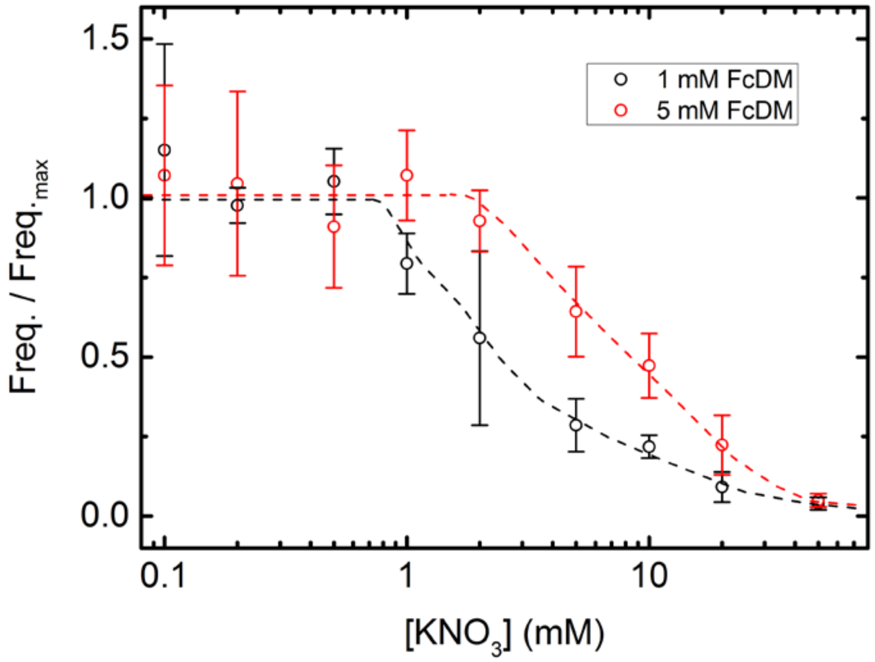

Figure S2. Frequency of collision measured with a 0.1 µg/mL GO solution. The black and red points correspond to a concentration of redox reporter (ferrocene dimethanol) of 1 mM and 5 mM, respectively. The error bars represent the SD between three measurements. The dashed lines are guides to the eye.

The existence of a plateau at low salt concentrations is intriguing since it is not captured by the PNP model. Possible causes for the appearance of a plateau were tested. First, we ruled out electrostatic repulsion between sheets in solution and sheets adsorbed on the UME by limiting the number of collision per trace to less than 10 (or < 20% decrease of the steady state current) and polishing the UME between each *i-t* trace to ensure a clean surface for each run. Second, we checked by DLS that the GO dispersion is stable over the course of our experiment (about 2h) and thus that the sheet concentration is constant. Finally, we changed the concentration of the redox reporter from 1 mM to 5 mM. A shown in Figure S2 the position of the plateau changes, suggesting that the electric field in the vicinity of the electrode plays a role. To test the generality of this effect, we repeated these experiments with carboxylated polystyrene 1 µm diameter beads as a "model" blocker to ensure that the particular nature of the GO sheets is not the cause of the plateau. Again, we observed that the frequency of collision of polystyrene beads levels off when the salt concentration becomes comparable to that of the reporter. This behavior therefore appears to be general rather than related to the specific properties of GO.

In an attempt to get a better qualitative understanding of the origin of the cross-over between two regimes at a supporting electrolyte concentration comparable to that of the FcMeOH concentration, it is interesting to consider theoretically the potential and ion distributions near the electrode. We consider an analytical model at the level of the Nernst-Planck equation in the charge-neutrality

approximation.[1,2] To make the calculation tractable, we assume a shrouded hemispherical disk electrode rather than a shrouded disk, and neglect possible effects of convection.[3] This derivation is fully equivalent to that of Oldham.[2]

The model includes four concentration profiles

$c_{\mathrm{Red}}(r)$, the concentration of FcMeOH in the reduced form (neutral, $z = 0$).
$c_{\mathrm{Ox}}(r)$, the concentration of FcMeOH in the oxidized form ($z = +1$).
$c_{+}(r)$, the concentration of K^+ supporting electrolyte ions ($z = +1$).
$c_{-}(r)$, the concentration of KNO_3^- supporting electrolyte ions ($z = -1$).

as well as a fifth unknown function:

$V(r)$, the local electrostatic potential.

In addition, we define the following constants:

c_{salt}, the value of $c_{+}(r)$ and $c_{-}(r)$ in the bulk far from the electrode.
c^{0}_{Red}, the value of $c_{\mathrm{Red}}(r \to \infty)$ in the bulk far from the electrode.
D_i, the diffusion coefficients for the four species defined above (here *i* stands for Red, Ox, + and -).
$f = F/RT$, where F is the Faraday constant, R is the gas constant and T is the absolute temperature.

We assume that charge neutrality is satisfied locally. This assumption is valid at distances from the electrode surface that are greater than a few Debye lengths from the surface of the electrode. This is sufficient as a first approximation since the electrode radius (*a* = 5 μm) is much larger than the Debye length at the lowest ionic strengths investigated here ($\lambda_D \lesssim 30$ nm). In terms of the ion concentrations, neutrality implies that

$$c_{\mathrm{Ox}}(r) = c_{-}(r) - c_{+}(r) \qquad \text{(S1)}$$

Furthermore, all four concentrations obey the Nernst-Planck equation,

$$\frac{\partial c_i}{\partial t} = \frac{1}{r^2}\frac{\partial}{\partial r}\left(-r^2 D_i\left(\frac{\partial c_i}{\partial r} + z_i c_i f\frac{\partial V}{\partial r}\right)\right)$$

In the steady state, $\frac{\partial c_i}{\partial t} = 0$ and the Nernst-Planck equation simplifies to

$$-2\pi r^2 D_i\left(\frac{dc_i}{dr} + z_i c_i f\frac{dV}{dr}\right) = J_i \qquad \text{(S2)}$$

Here J_i is the total outward flux of species i in moles/second. The boundary conditions in the steady state (in addition to the constraint on $V(r \to \infty)$ can be written as

$$c_{\mathrm{Red}}(r \to \infty) = c^{0}_{\mathrm{Red}}$$
$$c_{\mathrm{Red}}(a) = 0$$
$$c_{\mathrm{Ox}}(r \to \infty) = 0$$

[1]C. Amatore, M. R. Deakin, R. M. Wightman, *Journal of Electroanalytical Chemistry and Interfacial Electrochemistry* **1987**, *225*, 49-63.
[2]K. B. Oldham, *Journal of Electroanalytical Chemistry and Interfacial Electrochemistry* **1988**, *250*, 1-21.
[3] M. F. Bento, L. Thouin, C. Amatore, *Journal of Electroanalytical Chemistry* **1998**, *446*, 91-105.

$$J_{\mathrm{Ox}} = -J_{\mathrm{Red}} \text{ (corresponding to Red} \rightarrow \text{Ox at } r = a)$$
$$c_+(r \rightarrow \infty) = c_{\mathrm{salt}}$$
$$J_+ = 0$$
$$c_-(r \rightarrow \infty) = c_{\mathrm{salt}}$$
$$J_- = 0$$
$$V(r \rightarrow \infty) = 0$$

- For the reduced species with $z = 0$, the potential $V(r)$ does not play a role and eq S2 is easily solved to yield the well-known solution for diffusion-limited transport to a hemispherical UME at high oxidizing overpotential,

$$c_{\mathrm{Red}}(r) = c_{\mathrm{Red}}^0\left(1 - \frac{a}{r}\right) \tag{S3}$$

with flux $J_{\mathrm{Red}} = -2\pi a D_{\mathrm{Red}} c_{\mathrm{Red}}^0$.

- For the supporting electrolyte species, $J_\pm = 0$ in the steady state and eq S2 reduces to the equilibrium Boltzmann distribution for charge species in an electrostatic potential $V(r)$,

$$c_\pm(r) = c_{\mathrm{salt}} e^{\mp f V(r)} \tag{S4}$$

- Combining eq S1 and eq S4 yields an expression linking $c_{\mathrm{Ox}}(r)$ and $V(r)$:

$$c_{\mathrm{Ox}}(r) = 2c_{\mathrm{salt}} \sinh f V(r) \tag{S5}$$

We can obtain a single equation for $V(r)$ by substituting eq S5 into eq S2 for the case $i = \mathrm{Ox}$:

$$-2\pi r^2 D_{\mathrm{Ox}}\left(\frac{d}{dr}(2c_{\mathrm{salt}} \sinh f V(r)) + 2f c_{\mathrm{salt}} \sinh f V(r) \frac{dV}{dr}\right) = J_{\mathrm{Ox}}$$

Combined with $J_{\mathrm{Ox}} = -J_{\mathrm{Red}} = 2\pi a D_{\mathrm{Red}} c_{\mathrm{Red}}^0$, this expression has the solution

$$V(r) = \frac{1}{f}\ln\left(1 + \frac{D_{\mathrm{Red}} c_{\mathrm{Red}}^0}{2D_{\mathrm{Ox}} c_{\mathrm{salt}}}\frac{a}{r}\right) \tag{S6}$$

Eq S6 corresponds to eq 30 in Oldham.[3] The corresponding electric field is

$$\mathcal{E}(r) = -\frac{dV(r)}{dr} = \frac{1}{fr}\left(1 + \frac{2D_{\mathrm{Ox}} c_{\mathrm{salt}} r}{D_{\mathrm{Red}} c_{\mathrm{Red}}^0 a}\right)^{-1} \tag{S7}$$

while the corresponding solutions for the charged species are

$$c_-(r) = c_{\mathrm{salt}}\left(1 + \frac{D_{\mathrm{Red}} c_{\mathrm{Red}}^0}{2D_{\mathrm{Ox}} c_{\mathrm{salt}}}\frac{a}{r}\right) \tag{S8}$$

$$c_+(r) = c_{\mathrm{salt}}\left(1 + \frac{D_{\mathrm{Red}} c_{\mathrm{Red}}^0}{2D_{\mathrm{Ox}} c_{\mathrm{salt}}}\frac{a}{r}\right)^{-1} \tag{S9}$$

$$c_{\mathrm{Ox}}(r) = c_{\mathrm{salt}}\left(1 + \frac{D_{\mathrm{Red}} c_{\mathrm{Red}}^0}{2D_{\mathrm{Ox}} c_{\mathrm{salt}}}\frac{a}{r}\right) - c_{\mathrm{salt}}\left(1 + \frac{D_{\mathrm{Red}} c_{\mathrm{Red}}^0}{2D_{\mathrm{Ox}} c_{\mathrm{salt}}}\frac{a}{r}\right)^{-1} \tag{S10}$$

Eq S8 reduce to eq 1 in the main text upon making the simplification that $D_{\mathrm{Ox}} \approx D_{\mathrm{Red}}$. Eq S7 reduces to eq 2 in the main text upon further substituting

$$\mathcal{E}_{\mathrm{supp}}(r) = \frac{D_{\mathrm{Red}} c_{\mathrm{Red}}^{0} a}{2 f D_{\mathrm{Ox}} c_{\mathrm{salt}} r^{2}} \tag{S11}$$

This expression for $\mathcal{E}_{\mathrm{supp}}(r)$ is derived from eq S7 in the limit $c_{\mathrm{salt}} \gg c_{\mathrm{Red}}^{0}$.

Finite-element Simulations

All the simulations are performed using COMSOL Multiphysics 5.1 and the “transport of diluted species” package. The simulation of migration combines the “transport of diluted species” and “electrostatic” packages. A computer with a 3.1 GHz CPU and 4 GB RAM was used.

1. Migration-Diffusion of GO sheets

For the sake of clarity we provide the full COMSOL report of the simulation:

Contents

1. Global Definitions 9
  1.1. Parameters 1 9
2. Component 1 10
  2.1. Definitions 10
  2.2. Geometry 1 11
  2.3. Electrostatics 12
  2.4. Transport of Diluted Species 26
  2.5. Mesh 1 53
3. Study 1 61
  3.1. Parametric Sweep 61
  3.2. Time Dependent 61
  3.3. Solver Configurations 61
4. Results 64
  4.1. Data Sets ..... **Error! Bookmark not defined.**
  4.2. Tables ..... **Error! Bookmark not defined.**
  4.3. Plot Groups 64

1 Global Definitions

Global settings

1.1 Parameters 1

Parameters

Name	Expression	Value	Description
a	5[um]	5E−6 m	electrode's radius
b	2*a	1E−5 m	glass sheath near electrode
c	30*a	1.5E−4 m	inner cell
d	100*a	5E−4 m	outer cell
R	8.314[J/K/mol]	8.314 J/(mol·K)	gas constant
T	298 [K]	298 K	temperature
F	96485 [C/mol]	96485 C/mol	Faraday's constant
Na	6.022E23 [mol^(-1)]	6.022E23 1/mol	Avogadro's number
zOx	1	1	charge of Ox
zRed	0	0	charge of Red
zAnion	-1	−1	charge of Anion
zCation	1	1	charge of Cation
zGO	-1200	−1200	charge of GO sheet
DFc	6.7E-6[cm^2/s]	6.7E−10 m²/s	diffusion coefficient of ferrocene methanol (from litterature)
DNO3	1.76[m^2/s]	1.76 m²/s	diffusion coefficient of nitrate (from litterature)
DK	1.96E-9[m^2/s]	1.96E−9 m²/s	diffusion coefficient of potassium (from litterature)
DGO	5E-13[m^2/s]	5E−13 m²/s	diffusion coefficient of graphene oxide sheet (from DLS)
Cb	0.001[mol/L]	1 mol/m³	bulk concentration of ferrocene methanol (exp. parameter)
Csalt	0.001[mol/L]	1 mol/m³	bulk concentration of KNO3 (exp. parameter)
CGO	30E-15[mol/L]	3E−11 mol/m³	bulk concentration of graphene oxide sheet (from freq. of col. at high salt & D_DLS)
muK	DK/(R*T)	7.911E−13 s·mol/kg	mobility of potassium (from N-E eq.)
muNO3	DNO3/(R*T)	7.1037E−4 s·mol/kg	mobility of nitrate (from N-E eq.)
muOx	DFc/(R*T)	2.7043E−13 s·mol/kg	mobility of ferrocene methanol (from N-E eq.)
muGO	DGO/(R*T)	2.0181E−16 s·mol/kg	mobility of GO sheet (from N-E eq.)
muGO_th	-muGO_DLS/(zGO*F)	2.1592E−16 s·mol/kg	mobility of GO sheet (DLS mobility + zGO)
muGO_DLS	2.5E-8[m^2/V/s]	2.5E−8 m²/(V·s)	mobility of GO sheet (from DLS)

2 Component 1

Component settings

Unit system	SI
Geometry shape order	automatic

2.1 Definitions

2.1.1 Variables

Variables 1

Selection

Geometric entity level	Boundary
Selection	Boundary 2

Name	Expression	Unit	Description
current	F*DFc*intop1(cOxz)	A	current
freq	Na*DGO*intop1(cGOz)	1/s	collision frequency

2.1.2 Component Couplings

Integration 1

Coupling type	Integration
Operator name	intop1

Source selection

Geometric entity level	Boundary
Selection	Boundary 2

2.1.3 Coordinate Systems

Boundary System 1

Coordinate system type	Boundary system
Tag	sys1

Coordinate names

First (t1)	Second (to)	Third (n)
t1	to	n

Settings

Description	Value
Create first tangent direction from	Global Cartesian

2.2 Geometry 1

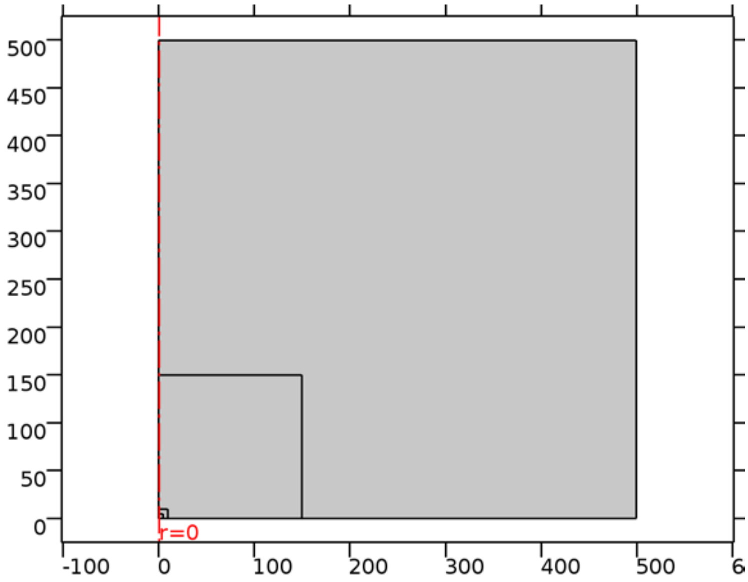

Geometry 1

Units

Length unit	µm
Angular unit	deg

Geometry statistics

Description	Value
Space dimension	2
Number of domains	5
Number of boundaries	19
Number of vertices	15

2.2.1 cell 1 (r5)

Position

Description	Value
Position	{0, 0}
Layers	

Size

Description	Value
Width	a
Height	a/20

2.2.2 cell 2 (r2)

Position

Description	Value
Position	{0, 0}
Layers	

Size

Description	Value

Width	a
Height	a

2.2.3 cell 3 (r3)

Position

Description	Value
Position	{0, 0}
Layers	

Size

Description	Value
Width	b
Height	b

2.2.4 cell 4 (r4)

Position

Description	Value
Position	{0, 0}
Layers	

Size

Description	Value
Width	c
Height	c

2.2.5 cell 5 (r6)

Position

Description	Value
Position	{0, 0}
Layers	

Size

Description	Value
Width	d
Height	d

2.3 Electrostatics

Used products

COMSOL Multiphysics

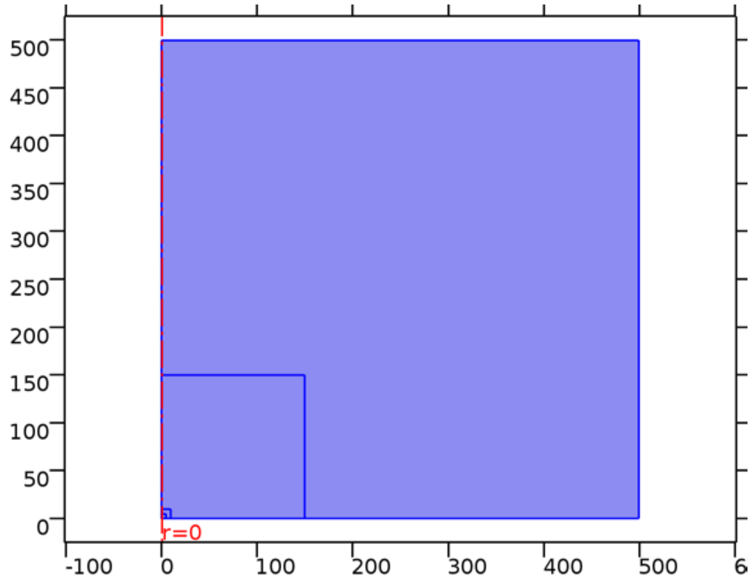

Electrostatics

Selection

Geometric entity level	Domain
Selection	Domains 1–5

Equations

$$\nabla \cdot \mathbf{D} = \rho_v$$

$$\mathbf{E} = -\nabla V$$

Settings

Description	Value
Electric potential	Quadratic
Value type when using splitting of complex variables	Complex
Activate terminal sweep	Off
Reference impedance	50[ohm]

Variables

Name	Expression	Unit	Description	Selection
es.d	1	1	Contribution	Domains 1–5
es.nr	nr		Normal vector, r component	Boundaries 4, 6, 8, 10, 12, 14–15, 17
es.nphi	0		Normal vector, phi component	Boundaries 4, 6, 8, 10, 12, 14–15, 17
es.nz	nz		Normal vector, z component	Boundaries 4, 6, 8, 10, 12, 14–15, 17
es.nr	dnr		Normal vector, r component	Boundaries 1–3, 5, 7, 9, 11, 13, 16, 18–19
es.nphi	0		Normal vector, phi component	Boundaries 1–3, 5, 7, 9, 11, 13, 16, 18–19

es.nz	dnz		Normal vector, z component	Boundaries 1–3, 5, 7, 9, 11, 13, 16, 18–19
es.nmeshr	nrmesh		Mesh normal vector, r component	Boundaries 4, 6, 8, 10, 12, 14–15, 17
es.nmeshphi	0		Mesh normal vector, phi component	Boundaries 4, 6, 8, 10, 12, 14–15, 17
es.nmeshz	nzmesh		Mesh normal vector, z component	Boundaries 4, 6, 8, 10, 12, 14–15, 17
es.nmeshr	dnrmesh		Mesh normal vector, r component	Boundaries 1–3, 5, 7, 9, 11, 13, 16, 18–19
es.nmeshphi	0		Mesh normal vector, phi component	Boundaries 1–3, 5, 7, 9, 11, 13, 16, 18–19
es.nmeshz	dnzmesh		Mesh normal vector, z component	Boundaries 1–3, 5, 7, 9, 11, 13, 16, 18–19
es.unmeshr	unrmesh		Mesh normal vector, upside, r component	Boundaries 4, 6, 8, 10, 12, 14–15, 17
es.unmeshphi	0		Mesh normal vector, upside, phi component	Boundaries 4, 6, 8, 10, 12, 14–15, 17
es.unmeshz	unzmesh		Mesh normal vector, upside, z component	Boundaries 4, 6, 8, 10, 12, 14–15, 17
es.dnmeshr	dnrmesh		Mesh normal vector, downside, r component	Boundaries 4, 6, 8, 10, 12, 14–15, 17
es.dnmeshphi	0		Mesh normal vector, downside, phi component	Boundaries 4, 6, 8, 10, 12, 14–15, 17
es.dnmeshz	dnzmesh		Mesh normal vector, downside, z component	Boundaries 4, 6, 8, 10, 12, 14–15, 17
es.unTer	0	Pa	Maxwell upward electric surface stress tensor, r component	Boundaries 1–19
es.unTephi	0	Pa	Maxwell upward electric surface stress tensor, phi component	Boundaries 1–19
es.unTez	0	Pa	Maxwell upward electric surface stress tensor, z component	Boundaries 1–19
es.dnTer	0	Pa	Maxwell downward electric surface stress tensor, r component	Boundaries 1–19
es.dnTephi	0	Pa	Maxwell downward electric surface stress tensor, phi component	Boundaries 1–19
es.dnTez	0	Pa	Maxwell downward electric surface stress tensor, z component	Boundaries 1–19
es.intWe	es.int_We(es.d *es.dWe)	J	Total electric energy	Global
es.zref	50[ohm]	Ω	Reference impedance	Global

2.3.1 Charge Conservation

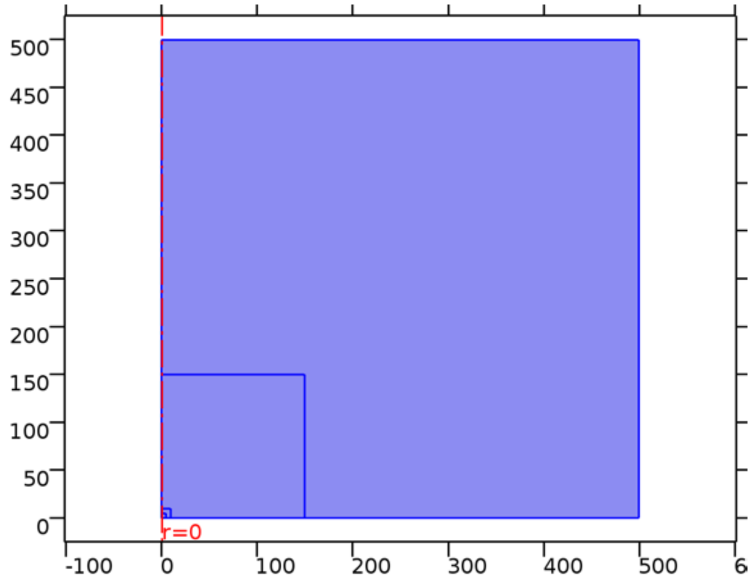

Charge Conservation

Selection

Geometric entity level	Domain
Selection	Domains 1–5

Equations

$$\mathbf{E} = -\nabla V$$
$$\nabla \cdot (\epsilon_0 \epsilon_r \mathbf{E}) = \rho_v$$

Settings

Description	Value
Constitutive relation	Relative permittivity
Relative permittivity	User defined
Relative permittivity	{{80, 0, 0}, {0, 80, 0}, {0, 0, 80}}

Variables

Name	Expression	Unit	Description	Selection
es.unTer	-0.5*es.dnr*(real(up(es.Dr))*real(up(es.Er))+real(up(es.Dphi))*real(up(es.Ephi))+real(up(es.Dz))*real(up(es.Ez)))+real(up(es.Dr))*(real(up(es.Er))*es.dnr+real(up(es.Ephi))*es.dnphi+real(up(es.Ez))*es.dnz)	Pa	Maxwell upward electric surface stress tensor, r component	Boundaries 4, 6, 8, 10, 12, 14–15, 17
es.unTephi	-0.5*es.dnphi*(real(up(es.Dr))*real(up(es.Er))+real(up(es.Dphi))*real(up(es.Ephi))+real(up(es.Dz))*real(up(es.Ez)))+real(up(es.Dphi))*(real(	Pa	Maxwell upward electric surface stress tensor, phi component	Boundaries 4, 6, 8, 10, 12, 14–15, 17

	up(es.Er))*es.dnr+real(up(es.Ephi))*es.dnphi+real(up(es.Ez))*es.dnz)			
es.unTez	-0.5*es.dnz*(real(up(es.Dr))*real(up(es.Er))+real(up(es.Dphi))*real(up(es.Ephi))+real(up(es.Dz))*real(up(es.Ez)))+real(up(es.Dz))*(real(up(es.Er))*es.dnr+real(up(es.Ephi))*es.dnphi+real(up(es.Ez))*es.dnz)	Pa	Maxwell upward electric surface stress tensor, z component	Boundaries 4, 6, 8, 10, 12, 14–15, 17
es.dnTer	-0.5*es.unr*(real(down(es.Dr))*real(down(es.Er))+real(down(es.Dphi))*real(down(es.Ephi))+real(down(es.Dz))*real(down(es.Ez)))+real(down(es.Dr))*(real(down(es.Er))*es.unr+real(down(es.Ephi))*es.unphi+real(down(es.Ez))*es.unz)	Pa	Maxwell downward electric surface stress tensor, r component	Boundaries 4, 6, 8, 10, 12, 14–15, 17
es.dnTephi	-0.5*es.unphi*(real(down(es.Dr))*real(down(es.Er))+real(down(es.Dphi))*real(down(es.Ephi))+real(down(es.Dz))*real(down(es.Ez)))+real(down(es.Dphi))*(real(down(es.Er))*es.unr+real(down(es.Ephi))*es.unphi+real(down(es.Ez))*es.unz)	Pa	Maxwell downward electric surface stress tensor, phi component	Boundaries 4, 6, 8, 10, 12, 14–15, 17
es.dnTez	-0.5*es.unz*(real(down(es.Dr))*real(down(es.Er))+real(down(es.Dphi))*real(down(es.Ephi))+real(down(es.Dz))*real(down(es.Ez)))+real(down(es.Dz))*(real(down(es.Er))*es.unr+real(down(es.Ephi))*es.unphi+real(down(es.Ez))*es.unz)	Pa	Maxwell downward electric surface stress tensor, z component	Boundaries 4, 6, 8, 10, 12, 14–15, 17
es.dnTer	-0.5*es.unr*(real(down(es.Dr))*real(down(es.Er))+real(down(es.Dphi))*real(down(es.Ephi))+real(down(es.Dz))*real(down(es.Ez)))+real(down(es.Dr))*(real(down(es.Er))*es.unr+real(down(es.Ep	Pa	Maxwell downward electric surface stress tensor, r component	Boundaries 1–3, 5, 7, 9, 11, 13, 16, 18–19

	hi))*es.unphi+real(down(es.Ez))*es.unz)			
es.dnTephi	-0.5*es.unphi*(real(down(es.Dr))*real(down(es.Er))+real(down(es.Dphi))*real(down(es.Ephi))+real(down(es.Dz))*real(down(es.Ez)))+real(down(es.Dphi))*(real(down(es.Er))*es.unr+real(down(es.Ephi))*es.unphi+real(down(es.Ez))*es.unz)	Pa	Maxwell downward electric surface stress tensor, phi component	Boundaries 1–3, 5, 7, 9, 11, 13, 16, 18–19
es.dnTez	-0.5*es.unz*(real(down(es.Dr))*real(down(es.Er))+real(down(es.Dphi))*real(down(es.Ephi))+real(down(es.Dz))*real(down(es.Ez)))+real(down(es.Dz))*(real(down(es.Er))*es.unr+real(down(es.Ephi))*es.unphi+real(down(es.Ez))*es.unz)	Pa	Maxwell downward electric surface stress tensor, z component	Boundaries 1–3, 5, 7, 9, 11, 13, 16, 18–19
es.nD	0	C/m^2	Surface charge density	Boundaries 1–19
es.epsilonrrr	80	1	Relative permittivity, rr component	Domains 1–5
es.epsilonrphir	0	1	Relative permittivity, phir component	Domains 1–5
es.epsilonrzr	0	1	Relative permittivity, zr component	Domains 1–5
es.epsilonrrphi	0	1	Relative permittivity, rphi component	Domains 1–5
es.epsilonrphiphi	80	1	Relative permittivity, phiphi component	Domains 1–5
es.epsilonrzphi	0	1	Relative permittivity, zphi component	Domains 1–5
es.epsilonrrz	0	1	Relative permittivity, rz component	Domains 1–5
es.epsilonrphiz	0	1	Relative permittivity, phiz component	Domains 1–5

es.epsilonrzz	80	1	Relative permittivity, zz component	Domains 1–5
es.Dr	epsilon0_const*es.Irr*es.Er+epsilon0_const*es.Irphi*es.Ephi+epsilon0_const*es.Irz*es.Ez+es.Pr	C/m^2	Electric displacement field, r component	Domains 1–5
es.Dphi	epsilon0_const*es.Iphir*es.Er+epsilon0_const*es.Iphiphi*es.Ephi+epsilon0_const*es.Iphiz*es.Ez+es.Pphi	C/m^2	Electric displacement field, phi component	Domains 1–5
es.Dz	epsilon0_const*es.Izr*es.Er+epsilon0_const*es.Izphi*es.Ephi+epsilon0_const*es.Izz*es.Ez+es.Pz	C/m^2	Electric displacement field, z component	Domains 1–5
es.Pr	epsilon0_const*(es.chirr*es.Er+es.chirphi*es.Ephi+es.chirz*es.Ez)	C/m^2	Polarization, r component	Domains 1–5
es.Pphi	epsilon0_const*(es.chiphir*es.Er+es.chiphiphi*es.Ephi+es.chiphiz*es.Ez)	C/m^2	Polarization, phi component	Domains 1–5
es.Pz	epsilon0_const*(es.chizr*es.Er+es.chizphi*es.Ephi+es.chizz*es.Ez)	C/m^2	Polarization, z component	Domains 1–5
es.normD	sqrt(realdot(es.Dr,es.Dr)+realdot(es.Dphi,es.Dphi)+realdot(es.Dz,es.Dz))	C/m^2	Electric displacement field norm	Domains 1–5
es.normP	sqrt(realdot(es.Pr,es.Pr)+realdot(es.Pphi,es.Pphi)+realdot(es.Pz,es.Pz))	C/m^2	Polarization norm	Domains 1–5
es.chirr	-1+es.epsilonrrr	1	Electric susceptibility, rr component	Domains 1–5
es.chiphir	es.epsilonrphir	1	Electric susceptibility, phir component	Domains 1–5
es.chizr	es.epsilonrzr	1	Electric susceptibility, zr component	Domains 1–5
es.chirphi	es.epsilonrrphi	1	Electric susceptibility, rphi component	Domains 1–5
es.chiphiphi	-1+es.epsilonrphiphi	1	Electric susceptibility, phiphi component	Domains 1–5
es.chizphi	es.epsilonrzphi	1	Electric susceptibility, zphi component	Domains 1–5

es.chirz	es.epsilonrrz	1	Electric susceptibility, rz component	Domains 1–5
es.chiphiz	es.epsilonrphiz	1	Electric susceptibility, phiz component	Domains 1–5
es.chizz	-1+es.epsilonrzz	1	Electric susceptibility, zz component	Domains 1–5
es.Irr	1	1	Spatial identity matrix, rr component	Domains 1–5
es.Iphir	0	1	Spatial identity matrix, phir component	Domains 1–5
es.Izr	0	1	Spatial identity matrix, zr component	Domains 1–5
es.Irphi	0	1	Spatial identity matrix, rphi component	Domains 1–5
es.Iphiphi	1	1	Spatial identity matrix, phiphi component	Domains 1–5
es.Izphi	0	1	Spatial identity matrix, zphi component	Domains 1–5
es.Irz	0	1	Spatial identity matrix, rz component	Domains 1–5
es.Iphiz	0	1	Spatial identity matrix, phiz component	Domains 1–5
es.Izz	1	1	Spatial identity matrix, zz component	Domains 1–5
es.Er	-Vr	V/m	Electric field, r component	Domains 1–5
es.Ephi	0	V/m	Electric field, phi component	Domains 1–5
es.Ez	-Vz	V/m	Electric field, z component	Domains 1–5
es.tEr	-VTr	V/m	Tangential electric field, r component	Boundaries 1–19
es.tEphi	0	V/m	Tangential electric field, phi component	Boundaries 1–19
es.tEz	-VTz	V/m	Tangential electric field, z component	Boundaries 1–19

es.normE	sqrt(realdot(es.Er,es.Er)+realdot(es.Ephi,es.Ephi)+realdot(es.Ez,es.Ez))	V/m	Electric field norm	Domains 1–5
es.Jr	es.Jdr	A/m^2	Current density, r component	Domains 1–5
es.Jphi	es.Jdphi	A/m^2	Current density, phi component	Domains 1–5
es.Jz	es.Jdz	A/m^2	Current density, z component	Domains 1–5
es.Jdr	0	A/m^2	Displacement current density, r component	Domains 1–5
es.Jdphi	0	A/m^2	Displacement current density, phi component	Domains 1–5
es.Jdz	0	A/m^2	Displacement current density, z component	Domains 1–5
es.unTr	0	Pa	Maxwell upward surface stress tensor, r component	Boundaries 1–3, 5, 7, 9, 11, 13, 16, 18–19
es.unTphi	0	Pa	Maxwell upward surface stress tensor, phi component	Boundaries 1–3, 5, 7, 9, 11, 13, 16, 18–19
es.unTz	0	Pa	Maxwell upward surface stress tensor, z component	Boundaries 1–3, 5, 7, 9, 11, 13, 16, 18–19
es.unTr	es.unTer	Pa	Maxwell upward surface stress tensor, r component	Boundaries 4, 6, 8, 10, 12, 14–15, 17
es.unTphi	es.unTephi	Pa	Maxwell upward surface stress tensor, phi component	Boundaries 4, 6, 8, 10, 12, 14–15, 17
es.unTz	es.unTez	Pa	Maxwell upward surface stress tensor, z component	Boundaries 4, 6, 8, 10, 12, 14–15, 17
es.dnTr	es.dnTer	Pa	Maxwell downward surface stress tensor, r component	Boundaries 1–3, 5, 7, 9, 11, 13, 16, 18–19
es.dnTphi	es.dnTephi	Pa	Maxwell downward surface stress	Boundaries 1–3, 5, 7, 9, 11, 13, 16, 18–19

			tensor, phi component	
es.dnTz	es.dnTez	Pa	Maxwell downward surface stress tensor, z component	Boundaries 1–3, 5, 7, 9, 11, 13, 16, 18–19
es.dnTr	es.dnTer	Pa	Maxwell downward surface stress tensor, r component	Boundaries 4, 6, 8, 10, 12, 14–15, 17
es.dnTphi	es.dnTephi	Pa	Maxwell downward surface stress tensor, phi component	Boundaries 4, 6, 8, 10, 12, 14–15, 17
es.dnTz	es.dnTez	Pa	Maxwell downward surface stress tensor, z component	Boundaries 4, 6, 8, 10, 12, 14–15, 17
es.unr	unr		Normal vector up direction, r component	Boundaries 1–19
es.unphi	0		Normal vector up direction, phi component	Boundaries 1–19
es.unz	unz		Normal vector up direction, z component	Boundaries 1–19
es.dnr	dnr		Normal vector down direction, r component	Boundaries 1–19
es.dnphi	0		Normal vector down direction, phi component	Boundaries 1–19
es.dnz	dnz		Normal vector down direction, z component	Boundaries 1–19
es.W	es.We	J/m^3	Energy density	Domains 1–5
es.dWe	2*es.We*pi*r	J/m^2	Integrand for total electric energy	Domains 1–5
es.We	0.5*epsilon0_const*((es.epsilonrrr*es.Er+es.epsilonrrphi*es.Ephi+es.epsilonrrz*es.Ez)*es.Er+(es.epsilonrphir*es.Er+es.epsilonrphiphi*es.Ephi+es.epsilonrphiz*es.Ez)*es.Ephi+(es.epsilonrzr*es.	J/m^3	Electric energy density	Domains 1–5

	Er+es.epsilonrzphi*es.Ephi+ es.epsilonrzz*es.Ez)*es.Ez)			

Shape functions

Name	Shape function	Unit	Description	Shape frame	Selection
V	Lagrange (Quadratic)	V	Electric potential	Material	Domains 1–5

Weak expressions

Weak expression	Integration frame	Selection
-2*(es.Dr*test(Vr)+es.Dz*test(Vz))*es.d*pi*r	Material	Domains 1–5

2.3.2 Axial Symmetry 1

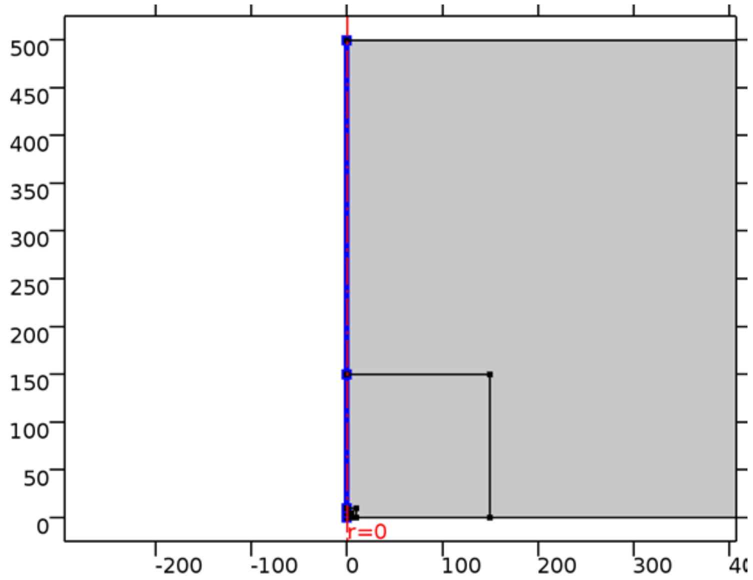

Axial Symmetry 1

Selection

Geometric entity level	Boundary
Selection	Boundaries 1, 3, 5, 7, 9

2.3.3 Zero Charge

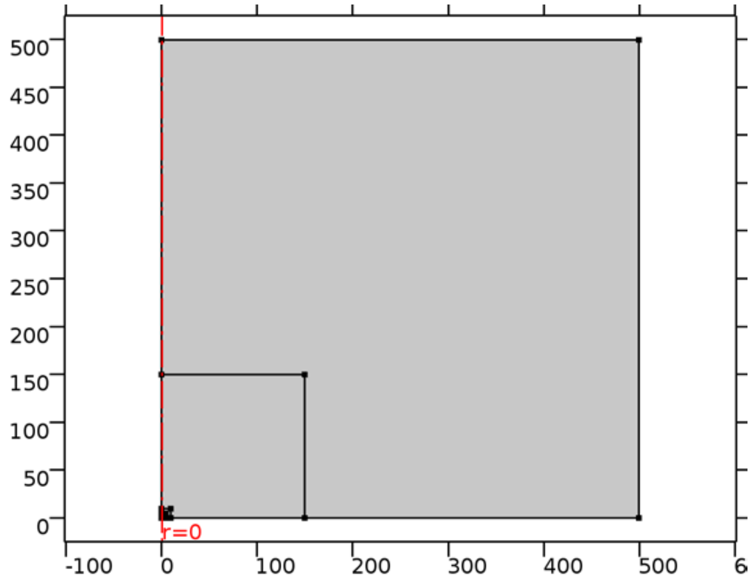

Zero Charge

Selection

Geometric entity level	Boundary
Selection	No boundaries

Equations

$$\mathbf{n} \cdot \mathbf{D} = 0$$

Shape functions

Name	Shape function	Unit	Description	Shape frame	Selection
V	Lagrange (Quadratic)	V	Electric potential	Material	No boundaries

2.3.4 Initial Values

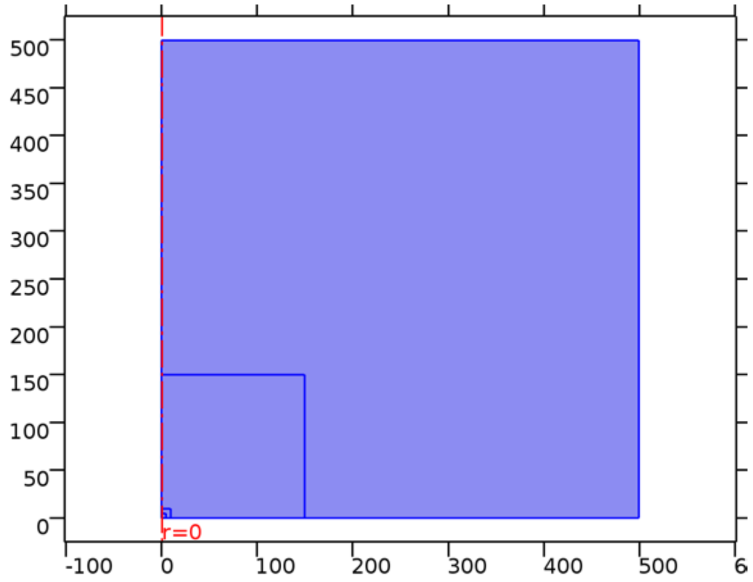

Initial Values

Selection

Geometric entity level	Domain
Selection	Domains 1–5

Settings

Description	Value
Electric potential	0

2.3.5 Space Charge Density

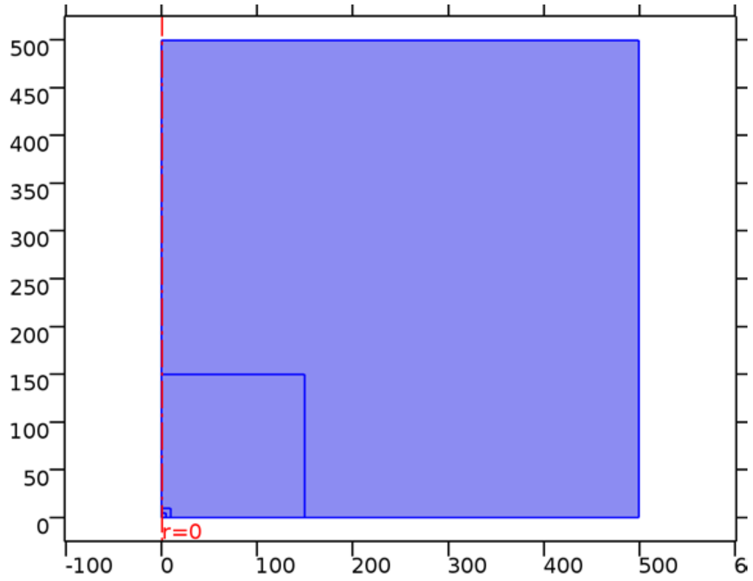

Space Charge Density

Selection

Geometric entity level	Domain
Selection	Domains 1–5

Equations

$$\nabla \cdot \mathbf{D} = \rho_v$$

Variables

Name	Expression	Unit	Description	Selection
es.scd1.rhoq	F*(zOx*cOx+zRed*cRed+zCation*cCation+zAnion*cAnion+zGO*cGO)	C/m^3	Space charge density	Domains 1–5
es.rhoq	es.scd1.rhoq	C/m^3	Space charge density	Domains 1–5

Weak expressions

Weak expression	Integration frame	Selection
-2*es.scd1.rhoq*test(V)*es.d*pi*r	Material	Domains 1–5

2.3.6 Electric Potential on solution boundary

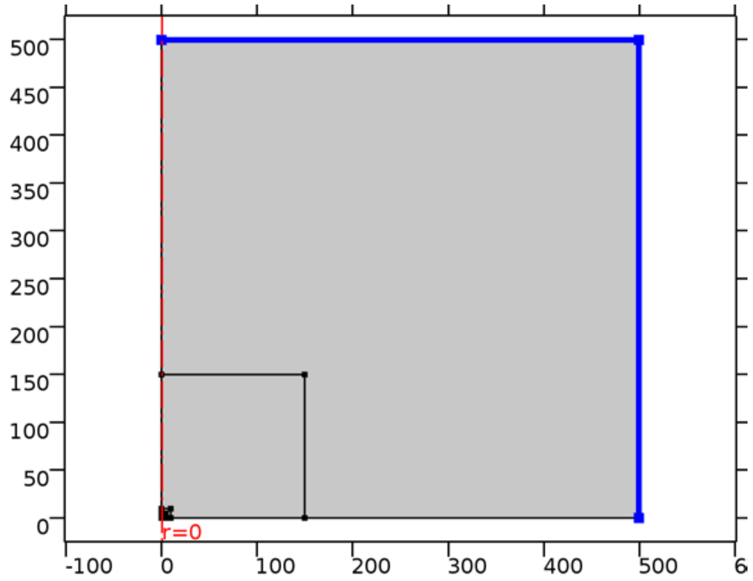

Electric Potential on solution boundary

Selection

Geometric entity level	Boundary
Selection	Boundaries 11, 19

Equations

$$V = V_0$$

Settings

Description	Value
Electric potential	0
Apply reaction terms on	All physics (symmetric)
Use weak constraints	Off
Constraint method	Elemental

Variables

Name	Expression	Unit	Description	Selection
es.nD	es.unr*(down(es.Dr)-up(es.Dr))+es.unphi*(down(es.Dphi)-up(es.Dphi))+es.unz*(down(es.Dz)-up(es.Dz))	C/m^2	Surface charge density	Boundaries 11, 19
es.V0	0	V	Electric potential	Boundaries 11, 19

Shape functions

Constraint	Constraint force	Shape function	Selection
es.V0-V	test(es.V0-V)	Lagrange (Quadratic)	Boundaries 11, 19

2.3.7 Surface Charge Density on electrode and glass sheath

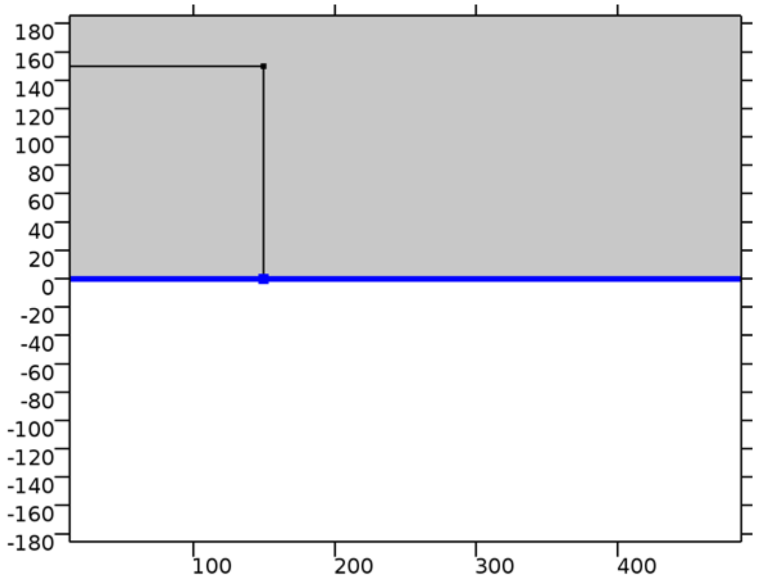

Surface Charge Density on electrode and glass sheath

Selection

Geometric entity level	Boundary
Selection	Boundaries 2, 13, 16, 18

Equations

$$\mathbf{n} \cdot (\mathbf{D}_1 - \mathbf{D}_2) = \rho_s$$

Settings

Description	Value
Surface charge density	0

Variables

Name	Expression	Unit	Description	Selection
es.nD	es.sfcd1.rhoqs	C/m^2	Surface charge density	Boundaries 2, 13, 16, 18
es.sfcd1.rhoqs	0	C/m^2	Surface charge density	Boundaries 2, 13, 16, 18

Weak expressions

Weak expression	Integration frame	Selection
-2*es.sfcd1.rhoqs*test(V)*es.d*pi*r	Material	Boundaries 2, 13, 16, 18

2.4 Transport of Diluted Species

Used products

COMSOL Multiphysics
Chemical Reaction Engineering Module

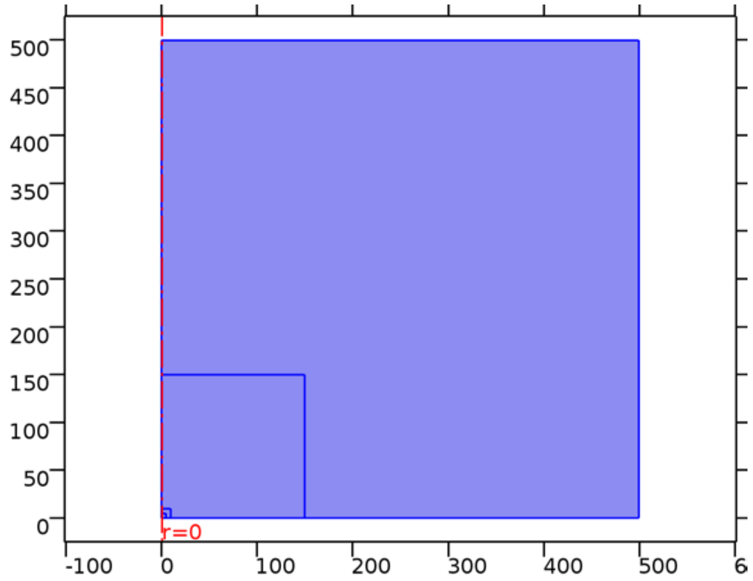

Transport of Diluted Species

Selection

Geometric entity level	Domain
Selection	Domains 1–5

Equations

$$\frac{\partial c_i}{\partial t} + \nabla \cdot (-D_i \nabla c_i - z_i u_{m,i} F c_i \nabla V) = R_i$$

$$\mathbf{N}_i = -D_i \nabla c_i - z_i u_{m,i} F c_i \nabla V$$

Settings

Description	Value
Concentration	Linear
Compute boundary fluxes	On
Apply smoothing to boundary fluxes	On
Value type when using splitting of complex variables	Real
Adsorption in porous media	Off
Dispersion in porous media	Off
Volatilization in partially saturated porous media	Off
Convection	Off
Migration in electric field	On
Streamline diffusion	On
Crosswind diffusion	On
Equation residual	Approximate residual
Crosswind diffusion type for free flow	Do Carmo and Galeão
Isotropic diffusion	Off
Convective term	Non - conservative form

Variables

Name	Expression	Unit	Description	Selection
domflux.cOxr	tds.dflux_cOxr+tds.mflux_cOxr	mol/(m^2*s)	Domain flux, r component	Domains 1–5

domflux.cOxz	tds.dflux_cOxz+tds.mflux_cOxz	mol/(m^2*s)	Domain flux, z component	Domains 1–5
domflux.cRedr	tds.dflux_cRedr+tds.mflux_cRedr	mol/(m^2*s)	Domain flux, r component	Domains 1–5
domflux.cRedz	tds.dflux_cRedz+tds.mflux_cRedz	mol/(m^2*s)	Domain flux, z component	Domains 1–5
domflux.cAnionr	tds.dflux_cAnionr+tds.mflux_cAnionr	mol/(m^2*s)	Domain flux, r component	Domains 1–5
domflux.cAnionz	tds.dflux_cAnionz+tds.mflux_cAnionz	mol/(m^2*s)	Domain flux, z component	Domains 1–5
domflux.cCationr	tds.dflux_cCationr+tds.mflux_cCationr	mol/(m^2*s)	Domain flux, r component	Domains 1–5
domflux.cCationz	tds.dflux_cCationz+tds.mflux_cCationz	mol/(m^2*s)	Domain flux, z component	Domains 1–5
domflux.cGOr	tds.dflux_cGOr+tds.mflux_cGOr	mol/(m^2*s)	Domain flux, r component	Domains 1–5
domflux.cGOz	tds.dflux_cGOz+tds.mflux_cGOz	mol/(m^2*s)	Domain flux, z component	Domains 1–5
tds.R_cOx	0	mol/(m^3*s)	Total rate expression	Domains 1–5
tds.R_cRed	0	mol/(m^3*s)	Total rate expression	Domains 1–5
tds.R_cAnion	0	mol/(m^3*s)	Total rate expression	Domains 1–5
tds.R_cCation	0	mol/(m^3*s)	Total rate expression	Domains 1–5
tds.R_cGO	0	mol/(m^3*s)	Total rate expression	Domains 1–5
tds.epsilon_p	1	1	Porosity	Domains 1–5
tds.theta	tds.epsilon_p	1	Liquid volume fraction	Domains 1–5
tds.av	0	1	Gas volume fraction	Domains 1–5
tds.nr	nr	1	Normal vector, r component	Boundaries 4, 6, 8, 10, 12, 14–15, 17
tds.nphi	0	1	Normal vector, phi component	Boundaries 4, 6, 8, 10, 12, 14–15, 17
tds.nz	nz	1	Normal vector, z component	Boundaries 4, 6, 8, 10, 12, 14–15, 17
tds.nr	dnr	1	Normal vector, r component	Boundaries 1–3, 5, 7, 9, 11, 13, 16, 18–19
tds.nphi	0	1	Normal vector, phi component	Boundaries 1–3, 5, 7, 9,

				11, 13, 16, 18–19
tds.nz	dnz	1	Normal vector, z component	Boundaries 1–3, 5, 7, 9, 11, 13, 16, 18–19
tds.nrmesh	nrmesh	1	Normal vector (mesh), r component	Boundaries 4, 6, 8, 10, 12, 14–15, 17
tds.nphimesh	0	1	Normal vector (mesh), phi component	Boundaries 4, 6, 8, 10, 12, 14–15, 17
tds.nzmesh	nzmesh	1	Normal vector (mesh), z component	Boundaries 4, 6, 8, 10, 12, 14–15, 17
tds.nrmesh	dnrmesh	1	Normal vector (mesh), r component	Boundaries 1–3, 5, 7, 9, 11, 13, 16, 18–19
tds.nphimesh	0	1	Normal vector (mesh), phi component	Boundaries 1–3, 5, 7, 9, 11, 13, 16, 18–19
tds.nzmesh	dnzmesh	1	Normal vector (mesh), z component	Boundaries 1–3, 5, 7, 9, 11, 13, 16, 18–19
tds.nrc	root.nrc/sqrt(root.nrc^2+root.nzc^2+eps)	1	Normal vector, r component	Boundaries 1–19
tds.nphic	0	1	Normal vector, phi component	Boundaries 1–19
tds.nzc	root.nzc/sqrt(root.nrc^2+root.nzc^2+eps)	1	Normal vector, z component	Boundaries 1–19
tds.ndflux_cOx	tds.dflux_cOxr*tds.nrc+tds.dflux_cOxphi*tds.nphic+tds.dflux_cOxz*tds.nzc	mol/(m^2*s)	Normal diffusive flux	Boundaries 2, 4, 6, 8, 10–19
tds.ndflux_cRed	tds.dflux_cRedr*tds.nrc+tds.dflux_cRedphi*tds.nphic+tds.dflux_cRedz*tds.nzc	mol/(m^2*s)	Normal diffusive flux	Boundaries 2, 4, 6, 8, 10–19
tds.ndflux_cAnion	tds.dflux_cAnionr*tds.nrc+tds.dflux_cAnionphi*tds.nphic+tds.dflux_cAnionz*tds.nzc	mol/(m^2*s)	Normal diffusive flux	Boundaries 2, 4, 6, 8, 10–19
tds.ndflux_cCation	tds.dflux_cCationr*tds.nrc+tds.dflux_cCationphi*tds.nphic+tds.	mol/(m^2*s)	Normal diffusive flux	Boundaries 2, 4, 6, 8, 10–19

	dflux_cCationz*tds.nzc			
tds.ndflux_cGO	tds.dflux_cGOr*tds.nrc+tds.dflux_cGOphi*tds.nphic+tds.dflux_cGOz*tds.nzc	mol/(m^2*s)	Normal diffusive flux	Boundaries 2, 4, 6, 8, 10–19
tds.nmflux_cOx	tds.mflux_cOxr*tds.nrc+tds.mflux_cOxphi*tds.nphic+tds.mflux_cOxz*tds.nzc	mol/(m^2*s)	Normal electrophoretic flux	Boundaries 2, 4, 6, 8, 10–19
tds.nmflux_cRed	tds.mflux_cRedr*tds.nrc+tds.mflux_cRedphi*tds.nphic+tds.mflux_cRedz*tds.nzc	mol/(m^2*s)	Normal electrophoretic flux	Boundaries 2, 4, 6, 8, 10–19
tds.nmflux_cAnion	tds.mflux_cAnionr*tds.nrc+tds.mflux_cAnionphi*tds.nphic+tds.mflux_cAnionz*tds.nzc	mol/(m^2*s)	Normal electrophoretic flux	Boundaries 2, 4, 6, 8, 10–19
tds.nmflux_cCation	tds.mflux_cCationr*tds.nrc+tds.mflux_cCationphi*tds.nphic+tds.mflux_cCationz*tds.nzc	mol/(m^2*s)	Normal electrophoretic flux	Boundaries 2, 4, 6, 8, 10–19
tds.nmflux_cGO	tds.mflux_cGOr*tds.nrc+tds.mflux_cGOphi*tds.nphic+tds.mflux_cGOz*tds.nzc	mol/(m^2*s)	Normal electrophoretic flux	Boundaries 2, 4, 6, 8, 10–19
tds.ntflux_cOx	tds.bndFlux_cOx	mol/(m^2*s)	Normal total flux	Boundaries 2, 4, 6, 8, 10–19
tds.ntflux_cRed	tds.bndFlux_cRed	mol/(m^2*s)	Normal total flux	Boundaries 2, 4, 6, 8, 10–19
tds.ntflux_cAnion	tds.bndFlux_cAnion	mol/(m^2*s)	Normal total flux	Boundaries 2, 4, 6, 8, 10–19
tds.ntflux_cCation	tds.bndFlux_cCation	mol/(m^2*s)	Normal total flux	Boundaries 2, 4, 6, 8, 10–19
tds.ntflux_cGO	tds.bndFlux_cGO	mol/(m^2*s)	Normal total flux	Boundaries 2, 4, 6, 8, 10–19
tds.bndFlux_cOx	if(r>0.001/sqrt(sqrt(mean(emetric2))),-0.5*dflux_spatial(cOx)/(pi*r),NaN)	mol/(m^2*s)	Boundary flux	Boundaries 1–3, 5, 7, 9, 11, 13, 16, 18–19
tds.bndFlux_cOx	if(r>0.001/sqrt(sqrt(mean(emetric2))),0.25*(uflux_spatial(cOx)-	mol/(m^2*s)	Boundary flux	Boundaries 4, 6, 8, 10, 12, 14–15, 17

	dflux_spatial(cOx))/(pi*r),NaN)			
tds.bndFlux_cRed	if(r>0.001/sqrt(sqrt(mean(emetric2))),-0.5*dflux_spatial(cRed)/(pi*r),NaN)	mol/(m^2*s)	Boundary flux	Boundaries 1–3, 5, 7, 9, 11, 13, 16, 18–19
tds.bndFlux_cRed	if(r>0.001/sqrt(sqrt(mean(emetric2))),0.25*(uflux_spatial(cRed)-dflux_spatial(cRed))/(pi*r),NaN)	mol/(m^2*s)	Boundary flux	Boundaries 4, 6, 8, 10, 12, 14–15, 17
tds.bndFlux_cAnion	if(r>0.001/sqrt(sqrt(mean(emetric2))),-0.5*dflux_spatial(cAnion)/(pi*r),NaN)	mol/(m^2*s)	Boundary flux	Boundaries 1–3, 5, 7, 9, 11, 13, 16, 18–19
tds.bndFlux_cAnion	if(r>0.001/sqrt(sqrt(mean(emetric2))),0.25*(uflux_spatial(cAnion)-dflux_spatial(cAnion))/(pi*r),NaN)	mol/(m^2*s)	Boundary flux	Boundaries 4, 6, 8, 10, 12, 14–15, 17
tds.bndFlux_cCation	if(r>0.001/sqrt(sqrt(mean(emetric2))),-0.5*dflux_spatial(cCation)/(pi*r),NaN)	mol/(m^2*s)	Boundary flux	Boundaries 1–3, 5, 7, 9, 11, 13, 16, 18–19
tds.bndFlux_cCation	if(r>0.001/sqrt(sqrt(mean(emetric2))),0.25*(uflux_spatial(cCation)-dflux_spatial(cCation))/(pi*r),NaN)	mol/(m^2*s)	Boundary flux	Boundaries 4, 6, 8, 10, 12, 14–15, 17
tds.bndFlux_cGO	if(r>0.001/sqrt(sqrt(mean(emetric2))),-0.5*dflux_spatial(cGO)/(pi*r),NaN)	mol/(m^2*s)	Boundary flux	Boundaries 1–3, 5, 7, 9, 11, 13, 16, 18–19
tds.bndFlux_cGO	if(r>0.001/sqrt(sqrt(mean(emetric2))),0.25*(uflux_spatial(cGO)-dflux_spatial(cGO))/(pi*r),NaN)	mol/(m^2*s)	Boundary flux	Boundaries 4, 6, 8, 10, 12, 14–15, 17

2.4.1 Transport Properties

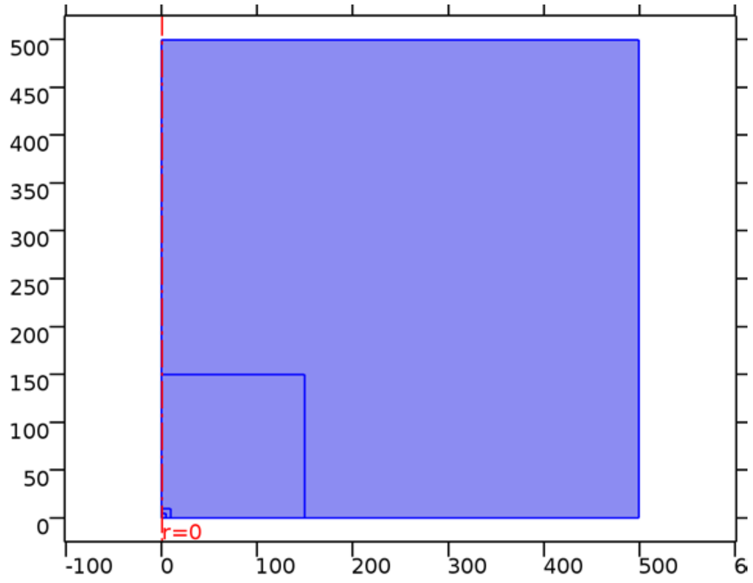

Transport Properties

Selection

Geometric entity level	Domain
Selection	Domains 1–5

Equations

$$\frac{\partial c_i}{\partial t} + \nabla \cdot (-D_i \nabla c_i - z_i u_{m,i} F c_i \nabla V) = R_i$$

$$\mathbf{N}_i = -D_i \nabla c_i - z_i u_{m,i} F c_i \nabla V$$

Settings

Description	Value
Electric potential	Electric potential (es)
Temperature	User defined
Temperature	293.15[K]
Material	None
Mobility	{{{muOx, 0, 0}, {0, muOx, 0}, {0, 0, muOx}}, {{muOx, 0, 0}, {0, muOx, 0}, {0, 0, muOx}}, {{muNO3, 0, 0}, {0, muNO3, 0}, {0, 0, muNO3}}, {{muK, 0, 0}, {0, muK, 0}, {0, 0, muK}}, {{muGO, 0, 0}, {0, muGO, 0}, {0, 0, muGO}}}
Charge number	{zOx, zRed, zAnion, zCation, zGO}
Mobility	User defined
Diffusion coefficient	User defined
Diffusion coefficient	{{DFc, 0, 0}, {0, DFc, 0}, {0, 0, DFc}}

Description	Value
Diffusion coefficient	User defined
Diffusion coefficient	{{DFc, 0, 0}, {0, DFc, 0}, {0, 0, DFc}}
Diffusion coefficient	User defined
Diffusion coefficient	{{DNO3, 0, 0}, {0, DNO3, 0}, {0, 0, DNO3}}
Diffusion coefficient	User defined
Diffusion coefficient	{{DK, 0, 0}, {0, DK, 0}, {0, 0, DK}}
Diffusion coefficient	User defined
Diffusion coefficient	{{DGO, 0, 0}, {0, DGO, 0}, {0, 0, DGO}}

Variables

Name	Expression	Unit	Description	Selection
tds.D_cOxrr	DFc	m^2/s	Diffusion coefficient, rr component	Domains 1–5
tds.D_cOxphir	0	m^2/s	Diffusion coefficient, phir component	Domains 1–5
tds.D_cOxzr	0	m^2/s	Diffusion coefficient, zr component	Domains 1–5
tds.D_cOxrphi	0	m^2/s	Diffusion coefficient, rphi component	Domains 1–5
tds.D_cOxphiphi	DFc	m^2/s	Diffusion coefficient, phiphi component	Domains 1–5
tds.D_cOxzphi	0	m^2/s	Diffusion coefficient, zphi component	Domains 1–5
tds.D_cOxrz	0	m^2/s	Diffusion coefficient, rz component	Domains 1–5
tds.D_cOxphiz	0	m^2/s	Diffusion coefficient, phiz component	Domains 1–5
tds.D_cOxzz	DFc	m^2/s	Diffusion coefficient, zz component	Domains 1–5
tds.D_cRedrr	DFc	m^2/s	Diffusion coefficient, rr component	Domains 1–5
tds.D_cRedphir	0	m^2/s	Diffusion coefficient, phir component	Domains 1–5
tds.D_cRedzr	0	m^2/s	Diffusion coefficient, zr component	Domains 1–5

Name	Expression	Unit	Description	Selection
tds.D_cRedrphi	0	m^2/s	Diffusion coefficient, rphi component	Domains 1–5
tds.D_cRedphiphi	DFc	m^2/s	Diffusion coefficient, phiphi component	Domains 1–5
tds.D_cRedzphi	0	m^2/s	Diffusion coefficient, zphi component	Domains 1–5
tds.D_cRedrz	0	m^2/s	Diffusion coefficient, rz component	Domains 1–5
tds.D_cRedphiz	0	m^2/s	Diffusion coefficient, phiz component	Domains 1–5
tds.D_cRedzz	DFc	m^2/s	Diffusion coefficient, zz component	Domains 1–5
tds.D_cAnionrr	DNO3	m^2/s	Diffusion coefficient, rr component	Domains 1–5
tds.D_cAnionphir	0	m^2/s	Diffusion coefficient, phir component	Domains 1–5
tds.D_cAnionzr	0	m^2/s	Diffusion coefficient, zr component	Domains 1–5
tds.D_cAnionrphi	0	m^2/s	Diffusion coefficient, rphi component	Domains 1–5
tds.D_cAnionphiphi	DNO3	m^2/s	Diffusion coefficient, phiphi component	Domains 1–5
tds.D_cAnionzphi	0	m^2/s	Diffusion coefficient, zphi component	Domains 1–5
tds.D_cAnionrz	0	m^2/s	Diffusion coefficient, rz component	Domains 1–5
tds.D_cAnionphiz	0	m^2/s	Diffusion coefficient, phiz component	Domains 1–5
tds.D_cAnionzz	DNO3	m^2/s	Diffusion coefficient, zz component	Domains 1–5
tds.D_cCationrr	DK	m^2/s	Diffusion coefficient, rr component	Domains 1–5
tds.D_cCationphir	0	m^2/s	Diffusion coefficient, phir component	Domains 1–5
tds.D_cCationzr	0	m^2/s	Diffusion coefficient, zr component	Domains 1–5

Name	Expression	Unit	Description	Selection
tds.D_cCationrphi	0	m^2/s	Diffusion coefficient, rphi component	Domains 1–5
tds.D_cCationphiphi	DK	m^2/s	Diffusion coefficient, phiphi component	Domains 1–5
tds.D_cCationzphi	0	m^2/s	Diffusion coefficient, zphi component	Domains 1–5
tds.D_cCationrz	0	m^2/s	Diffusion coefficient, rz component	Domains 1–5
tds.D_cCationphiz	0	m^2/s	Diffusion coefficient, phiz component	Domains 1–5
tds.D_cCationzz	DK	m^2/s	Diffusion coefficient, zz component	Domains 1–5
tds.D_cGOrr	DGO	m^2/s	Diffusion coefficient, rr component	Domains 1–5
tds.D_cGOphir	0	m^2/s	Diffusion coefficient, phir component	Domains 1–5
tds.D_cGOzr	0	m^2/s	Diffusion coefficient, zr component	Domains 1–5
tds.D_cGOrphi	0	m^2/s	Diffusion coefficient, rphi component	Domains 1–5
tds.D_cGOphiphi	DGO	m^2/s	Diffusion coefficient, phiphi component	Domains 1–5
tds.D_cGOzphi	0	m^2/s	Diffusion coefficient, zphi component	Domains 1–5
tds.D_cGOrz	0	m^2/s	Diffusion coefficient, rz component	Domains 1–5
tds.D_cGOphiz	0	m^2/s	Diffusion coefficient, phiz component	Domains 1–5
tds.D_cGOzz	DGO	m^2/s	Diffusion coefficient, zz component	Domains 1–5
tds.Dav_cOx	0.5*(tds.D_cOxrr+tds.D_cOxzz)	m^2/s	Average diffusion coefficient	Domains 1–5
tds.Dav_cRed	0.5*(tds.D_cRedrr+tds.D_cRedzz)	m^2/s	Average diffusion coefficient	Domains 1–5
tds.Dav_cAnion	0.5*(tds.D_cAnionrr+tds.D_cAnionzz)	m^2/s	Average diffusion coefficient	Domains 1–5

Name	Expression	Unit	Description	Selection
tds.Dav_cCation	0.5*(tds.D_cCationrr+tds.D_cCationzz)	m^2/s	Average diffusion coefficient	Domains 1–5
tds.Dav_cGO	0.5*(tds.D_cGOrr+tds.D_cGOzz)	m^2/s	Average diffusion coefficient	Domains 1–5
tds.tflux_cOxr	tds.dflux_cOxr+tds.mflux_cOxr	mol/(m^2*s)	Total flux, r component	Domains 1–5
tds.tflux_cOxphi	tds.dflux_cOxphi+tds.mflux_cOxphi	mol/(m^2*s)	Total flux, phi component	Domains 1–5
tds.tflux_cOxz	tds.dflux_cOxz+tds.mflux_cOxz	mol/(m^2*s)	Total flux, z component	Domains 1–5
tds.tflux_cRedr	tds.dflux_cRedr+tds.mflux_cRedr	mol/(m^2*s)	Total flux, r component	Domains 1–5
tds.tflux_cRedphi	tds.dflux_cRedphi+tds.mflux_cRedphi	mol/(m^2*s)	Total flux, phi component	Domains 1–5
tds.tflux_cRedz	tds.dflux_cRedz+tds.mflux_cRedz	mol/(m^2*s)	Total flux, z component	Domains 1–5
tds.tflux_cAnionr	tds.dflux_cAnionr+tds.mflux_cAnionr	mol/(m^2*s)	Total flux, r component	Domains 1–5
tds.tflux_cAnionphi	tds.dflux_cAnionphi+tds.mflux_cAnionphi	mol/(m^2*s)	Total flux, phi component	Domains 1–5
tds.tflux_cAnionz	tds.dflux_cAnionz+tds.mflux_cAnionz	mol/(m^2*s)	Total flux, z component	Domains 1–5
tds.tflux_cCationr	tds.dflux_cCationr+tds.mflux_cCationr	mol/(m^2*s)	Total flux, r component	Domains 1–5
tds.tflux_cCationphi	tds.dflux_cCationphi+tds.mflux_cCationphi	mol/(m^2*s)	Total flux, phi component	Domains 1–5
tds.tflux_cCationz	tds.dflux_cCationz+tds.mflux_cCationz	mol/(m^2*s)	Total flux, z component	Domains 1–5
tds.tflux_cGOr	tds.dflux_cGOr+tds.mflux_cGOr	mol/(m^2*s)	Total flux, r component	Domains 1–5
tds.tflux_cGOphi	tds.dflux_cGOphi+tds.mflux_cGOphi	mol/(m^2*s)	Total flux, phi component	Domains 1–5
tds.tflux_cGOz	tds.dflux_cGOz+tds.mflux_cGOz	mol/(m^2*s)	Total flux, z component	Domains 1–5

Name	Expression	Unit	Description	Selection
tds.dfluxMag_cOx	sqrt(tds.dflux_cOxr^2+tds.dflux_cOxphi^2+tds.dflux_cOxz^2)	mol/(m^2*s)	Diffusive flux magnitude	Domains 1–5
tds.dfluxMag_cRed	sqrt(tds.dflux_cRedr^2+tds.dflux_cRedphi^2+tds.dflux_cRedz^2)	mol/(m^2*s)	Diffusive flux magnitude	Domains 1–5
tds.dfluxMag_cAnion	sqrt(tds.dflux_cAnionr^2+tds.dflux_cAnionphi^2+tds.dflux_cAnionz^2)	mol/(m^2*s)	Diffusive flux magnitude	Domains 1–5
tds.dfluxMag_cCation	sqrt(tds.dflux_cCationr^2+tds.dflux_cCationphi^2+tds.dflux_cCationz^2)	mol/(m^2*s)	Diffusive flux magnitude	Domains 1–5
tds.dfluxMag_cGO	sqrt(tds.dflux_cGOr^2+tds.dflux_cGOphi^2+tds.dflux_cGOz^2)	mol/(m^2*s)	Diffusive flux magnitude	Domains 1–5
tds.tfluxMag_cOx	sqrt(tds.tflux_cOxr^2+tds.tflux_cOxphi^2+tds.tflux_cOxz^2)	mol/(m^2*s)	Total flux magnitude	Domains 1–5
tds.tfluxMag_cRed	sqrt(tds.tflux_cRedr^2+tds.tflux_cRedphi^2+tds.tflux_cRedz^2)	mol/(m^2*s)	Total flux magnitude	Domains 1–5
tds.tfluxMag_cAnion	sqrt(tds.tflux_cAnionr^2+tds.tflux_cAnionphi^2+tds.tflux_cAnionz^2)	mol/(m^2*s)	Total flux magnitude	Domains 1–5
tds.tfluxMag_cCation	sqrt(tds.tflux_cCationr^2+tds.tflux_cCationphi^2+tds.tflux_cCationz^2)	mol/(m^2*s)	Total flux magnitude	Domains 1–5
tds.tfluxMag_cGO	sqrt(tds.tflux_cGOr^2+tds.tflux_cGOphi^2+tds.tflux_cGOz^2)	mol/(m^2*s)	Total flux magnitude	Domains 1–5
tds.mflux_cOxr	tds.z_cOx*F_const*cOx*(-tds.um_cOxrr*d(tds.V,r)-tds.um_cOxrz*d(tds.V,z))	mol/(m^2*s)	Electrophoretic flux, r component	Domains 1–5

Name	Expression	Unit	Description	Selection
tds.mflux_cOxphi	tds.z_cOx*F_const*cOx*(-tds.um_cOxphir*d(tds.V,r)-tds.um_cOxphiz*d(tds.V,z))	mol/(m^2*s)	Electrophoretic flux, phi component	Domains 1–5
tds.mflux_cOxz	tds.z_cOx*F_const*cOx*(-tds.um_cOxzr*d(tds.V,r)-tds.um_cOxzz*d(tds.V,z))	mol/(m^2*s)	Electrophoretic flux, z component	Domains 1–5
tds.mflux_cRedr	tds.z_cRed*F_const*cRed*(-tds.um_cRedrr*d(tds.V,r)-tds.um_cRedrz*d(tds.V,z))	mol/(m^2*s)	Electrophoretic flux, r component	Domains 1–5
tds.mflux_cRedphi	tds.z_cRed*F_const*cRed*(-tds.um_cRedphir*d(tds.V,r)-tds.um_cRedphiz*d(tds.V,z))	mol/(m^2*s)	Electrophoretic flux, phi component	Domains 1–5
tds.mflux_cRedz	tds.z_cRed*F_const*cRed*(-tds.um_cRedzr*d(tds.V,r)-tds.um_cRedzz*d(tds.V,z))	mol/(m^2*s)	Electrophoretic flux, z component	Domains 1–5
tds.mflux_cAnionr	tds.z_cAnion*F_const*cAnion*(-tds.um_cAnionrr*d(tds.V,r)-tds.um_cAnionrz*d(tds.V,z))	mol/(m^2*s)	Electrophoretic flux, r component	Domains 1–5
tds.mflux_cAnionphi	tds.z_cAnion*F_const*cAnion*(-tds.um_cAnionphir*d(tds.V,r)-tds.um_cAnionphiz*d(tds.V,z))	mol/(m^2*s)	Electrophoretic flux, phi component	Domains 1–5

Name	Expression	Unit	Description	Selection
tds.mflux_cAnionz	tds.z_cAnion*F_const*cAnion*(-tds.um_cAnionzr*d(tds.V,r)-tds.um_cAnionzz*d(tds.V,z))	mol/(m^2*s)	Electrophoretic flux, z component	Domains 1–5
tds.mflux_cCationr	tds.z_cCation*F_const*cCation*(-tds.um_cCationrr*d(tds.V,r)-tds.um_cCationrz*d(tds.V,z))	mol/(m^2*s)	Electrophoretic flux, r component	Domains 1–5
tds.mflux_cCationphi	tds.z_cCation*F_const*cCation*(-tds.um_cCationphir*d(tds.V,r)-tds.um_cCationphiz*d(tds.V,z))	mol/(m^2*s)	Electrophoretic flux, phi component	Domains 1–5
tds.mflux_cCationz	tds.z_cCation*F_const*cCation*(-tds.um_cCationzr*d(tds.V,r)-tds.um_cCationzz*d(tds.V,z))	mol/(m^2*s)	Electrophoretic flux, z component	Domains 1–5
tds.mflux_cGOr	tds.z_cGO*F_const*cGO*(-tds.um_cGOrr*d(tds.V,r)-tds.um_cGOrz*d(tds.V,z))	mol/(m^2*s)	Electrophoretic flux, r component	Domains 1–5
tds.mflux_cGOphi	tds.z_cGO*F_const*cGO*(-tds.um_cGOphir*d(tds.V,r)-tds.um_cGOphiz*d(tds.V,z))	mol/(m^2*s)	Electrophoretic flux, phi component	Domains 1–5
tds.mflux_cGOz	tds.z_cGO*F_const*cGO*(-tds.um_cGOzr*d(tds.V,r)-tds.um_cGOzz*d(tds.V,z))	mol/(m^2*s)	Electrophoretic flux, z component	Domains 1–5

Name	Expression	Unit	Description	Selection
tds.dflux_cOxr	-tds.D_cOxrr*cOxr-tds.D_cOxrz*cOxz	mol/(m^2*s)	Diffusive flux, r component	Domains 1–5
tds.dflux_cOxphi	-tds.D_cOxphir*cOxr-tds.D_cOxphiz*cOxz	mol/(m^2*s)	Diffusive flux, phi component	Domains 1–5
tds.dflux_cOxz	-tds.D_cOxzr*cOxr-tds.D_cOxzz*cOxz	mol/(m^2*s)	Diffusive flux, z component	Domains 1–5
tds.dflux_cRedr	-tds.D_cRedrr*cRedr-tds.D_cRedrz*cRedz	mol/(m^2*s)	Diffusive flux, r component	Domains 1–5
tds.dflux_cRedphi	-tds.D_cRedphir*cRedr-tds.D_cRedphiz*cRedz	mol/(m^2*s)	Diffusive flux, phi component	Domains 1–5
tds.dflux_cRedz	-tds.D_cRedzr*cRedr-tds.D_cRedzz*cRedz	mol/(m^2*s)	Diffusive flux, z component	Domains 1–5
tds.dflux_cAnionr	-tds.D_cAnionrr*cAnionr-tds.D_cAnionrz*cAnionz	mol/(m^2*s)	Diffusive flux, r component	Domains 1–5
tds.dflux_cAnionphi	-tds.D_cAnionphir*cAnionr-tds.D_cAnionphiz*cAnionz	mol/(m^2*s)	Diffusive flux, phi component	Domains 1–5
tds.dflux_cAnionz	-tds.D_cAnionzr*cAnionr-tds.D_cAnionzz*cAnionz	mol/(m^2*s)	Diffusive flux, z component	Domains 1–5
tds.dflux_cCationr	-tds.D_cCationrr*cCationr-tds.D_cCationrz*cCationz	mol/(m^2*s)	Diffusive flux, r component	Domains 1–5
tds.dflux_cCationphi	-tds.D_cCationphir*cCationr-tds.D_cCationphiz*cCationz	mol/(m^2*s)	Diffusive flux, phi component	Domains 1–5

Name	Expression	Unit	Description	Selection
tds.dflux_cCationz	-tds.D_cCationzr*cCationr-tds.D_cCationzz*cCationz	mol/(m^2*s)	Diffusive flux, z component	Domains 1–5
tds.dflux_cGOr	-tds.D_cGOrr*cGOr-tds.D_cGOrz*cGOz	mol/(m^2*s)	Diffusive flux, r component	Domains 1–5
tds.dflux_cGOphi	-tds.D_cGOphir*cGOr-tds.D_cGOphiz*cGOz	mol/(m^2*s)	Diffusive flux, phi component	Domains 1–5
tds.dflux_cGOz	-tds.D_cGOzr*cGOr-tds.D_cGOzz*cGOz	mol/(m^2*s)	Diffusive flux, z component	Domains 1–5
tds.grad_cOxr	cOxr	mol/m^4	Concentration gradient, r component	Domains 1–5
tds.grad_cOxphi	0	mol/m^4	Concentration gradient, phi component	Domains 1–5
tds.grad_cOxz	cOxz	mol/m^4	Concentration gradient, z component	Domains 1–5
tds.grad_cRedr	cRedr	mol/m^4	Concentration gradient, r component	Domains 1–5
tds.grad_cRedphi	0	mol/m^4	Concentration gradient, phi component	Domains 1–5
tds.grad_cRedz	cRedz	mol/m^4	Concentration gradient, z component	Domains 1–5
tds.grad_cAnionr	cAnionr	mol/m^4	Concentration gradient, r component	Domains 1–5
tds.grad_cAnionphi	0	mol/m^4	Concentration gradient, phi component	Domains 1–5
tds.grad_cAnionz	cAnionz	mol/m^4	Concentration gradient, z component	Domains 1–5

Name	Expression	Unit	Description	Selection
tds.grad_cCationr	cCationr	mol/m^4	Concentration gradient, r component	Domains 1–5
tds.grad_cCationphi	0	mol/m^4	Concentration gradient, phi component	Domains 1–5
tds.grad_cCationz	cCationz	mol/m^4	Concentration gradient, z component	Domains 1–5
tds.grad_cGOr	cGOr	mol/m^4	Concentration gradient, r component	Domains 1–5
tds.grad_cGOphi	0	mol/m^4	Concentration gradient, phi component	Domains 1–5
tds.grad_cGOz	cGOz	mol/m^4	Concentration gradient, z component	Domains 1–5
tds.um_cOxrr	muOx	s*mol/kg	Mobility, rr component	Domains 1–5
tds.um_cOxphir	0	s*mol/kg	Mobility, phir component	Domains 1–5
tds.um_cOxzr	0	s*mol/kg	Mobility, zr component	Domains 1–5
tds.um_cOxrphi	0	s*mol/kg	Mobility, rphi component	Domains 1–5
tds.um_cOxphiphi	muOx	s*mol/kg	Mobility, phiphi component	Domains 1–5
tds.um_cOxzphi	0	s*mol/kg	Mobility, zphi component	Domains 1–5
tds.um_cOxrz	0	s*mol/kg	Mobility, rz component	Domains 1–5
tds.um_cOxphiz	0	s*mol/kg	Mobility, phiz component	Domains 1–5
tds.um_cOxzz	muOx	s*mol/kg	Mobility, zz component	Domains 1–5

Name	Expression	Unit	Description		Selection
tds.um_cRedrr	muOx	s*mol/kg	Mobility, component	rr	Domains 1–5
tds.um_cRedphir	0	s*mol/kg	Mobility, component	phir	Domains 1–5
tds.um_cRedzr	0	s*mol/kg	Mobility, component	zr	Domains 1–5
tds.um_cRedrphi	0	s*mol/kg	Mobility, component	rphi	Domains 1–5
tds.um_cRedphiphi	muOx	s*mol/kg	Mobility, component	phiphi	Domains 1–5
tds.um_cRedzphi	0	s*mol/kg	Mobility, component	zphi	Domains 1–5
tds.um_cRedrz	0	s*mol/kg	Mobility, component	rz	Domains 1–5
tds.um_cRedphiz	0	s*mol/kg	Mobility, component	phiz	Domains 1–5
tds.um_cRedzz	muOx	s*mol/kg	Mobility, component	zz	Domains 1–5
tds.um_cAnionrr	muNO3	s*mol/kg	Mobility, component	rr	Domains 1–5
tds.um_cAnionphir	0	s*mol/kg	Mobility, component	phir	Domains 1–5
tds.um_cAnionzr	0	s*mol/kg	Mobility, component	zr	Domains 1–5
tds.um_cAnionrphi	0	s*mol/kg	Mobility, component	rphi	Domains 1–5
tds.um_cAnionphiphi	muNO3	s*mol/kg	Mobility, component	phiphi	Domains 1–5
tds.um_cAnionzphi	0	s*mol/kg	Mobility, component	zphi	Domains 1–5
tds.um_cAnionrz	0	s*mol/kg	Mobility, component	rz	Domains 1–5
tds.um_cAnionphiz	0	s*mol/kg	Mobility, component	phiz	Domains 1–5
tds.um_cAnionzz	muNO3	s*mol/kg	Mobility, component	zz	Domains 1–5

Name	Expression	Unit	Description	Selection
tds.um_cCationrr	muK	s*mol/kg	Mobility, component rr	Domains 1–5
tds.um_cCationphir	0	s*mol/kg	Mobility, component phir	Domains 1–5
tds.um_cCationzr	0	s*mol/kg	Mobility, component zr	Domains 1–5
tds.um_cCationrphi	0	s*mol/kg	Mobility, component rphi	Domains 1–5
tds.um_cCationphiphi	muK	s*mol/kg	Mobility, component phiphi	Domains 1–5
tds.um_cCationzphi	0	s*mol/kg	Mobility, component zphi	Domains 1–5
tds.um_cCationrz	0	s*mol/kg	Mobility, component rz	Domains 1–5
tds.um_cCationphiz	0	s*mol/kg	Mobility, component phiz	Domains 1–5
tds.um_cCationzz	muK	s*mol/kg	Mobility, component zz	Domains 1–5
tds.um_cGOrr	muGO	s*mol/kg	Mobility, component rr	Domains 1–5
tds.um_cGOphir	0	s*mol/kg	Mobility, component phir	Domains 1–5
tds.um_cGOzr	0	s*mol/kg	Mobility, component zr	Domains 1–5
tds.um_cGOrphi	0	s*mol/kg	Mobility, component rphi	Domains 1–5
tds.um_cGOphiphi	muGO	s*mol/kg	Mobility, component phiphi	Domains 1–5
tds.um_cGOzphi	0	s*mol/kg	Mobility, component zphi	Domains 1–5
tds.um_cGOrz	0	s*mol/kg	Mobility, component rz	Domains 1–5
tds.um_cGOphiz	0	s*mol/kg	Mobility, component phiz	Domains 1–5
tds.um_cGOzz	muGO	s*mol/kg	Mobility, component zz	Domains 1–5

Name	Expression	Unit	Description	Selection
tds.z_cOx	zOx	1	Charge number	Domains 1–5
tds.z_cRed	zRed	1	Charge number	Domains 1–5
tds.z_cAnion	zAnion	1	Charge number	Domains 1–5
tds.z_cCation	zCation	1	Charge number	Domains 1–5
tds.z_cGO	zGO	1	Charge number	Domains 1–5
tds.V	model.input.V	V	Electric potential	Domains 1–5
tds.Res_cOx	cOxt+d(cOx*tds.z_cOx*F_const*(-tds.um_cOxrr*d(tds.V,r)-tds.um_cOxrz*d(tds.V,z)),r)+if(abs(r)<0.001*h,d(cOx*tds.z_cOx*F_const*(-tds.um_cOxrr*d(tds.V,r)-tds.um_cOxrz*d(tds.V,z)),r),cOx*tds.z_cOx*F_const*(-tds.um_cOxrr*d(tds.V,r)-tds.um_cOxrz*d(tds.V,z))/r)+d(cOx*tds.z_cOx*F_const*(-tds.um_cOxzr*d(tds.V,r)-tds.um_cOxzz*d(tds.V,z)),z)-tds.R_cOx	mol/(m^3*s)	Equation residual	Domains 1–5
tds.Res_cRed	cRedt+d(cRed*tds.z_cRed*F_const*(-tds.um_cRedrr*d(tds.V,r)-tds.um_cRedrz*d(tds.V,z)),r)+if(abs(r)<0.001*h,d(cRed*tds.z_cRed*F_const*(-tds.um_cRedrr*d(tds.V,r)-tds.um_cRedrz*d(tds.V,z)),r),cRed*tds.z_cRed*F_const*(-tds.um_cRedrr*d(tds.	mol/(m^3*s)	Equation residual	Domains 1–5

Name	Expression	Unit	Description	Selection
	V,r)-tds.um_cRedrz*d(tds.V,z))/r)+d(cRed*tds.z_cRed*F_const*(-tds.um_cRedzr*d(tds.V,r)-tds.um_cRedzz*d(tds.V,z)),z)-tds.R_cRed			
tds.Res_cAnion	cAniont+d(cAnion*tds.z_cAnion*F_const*(-tds.um_cAnionrr*d(tds.V,r)-tds.um_cAnionrz*d(tds.V,z)),r)+if(abs(r)<0.001*h,d(cAnion*tds.z_cAnion*F_const*(-tds.um_cAnionrr*d(tds.V,r)-tds.um_cAnionrz*d(tds.V,z)),r),cAnion*tds.z_cAnion*F_const*(-tds.um_cAnionrr*d(tds.V,r)-tds.um_cAnionrz*d(tds.V,z))/r)+d(cAnion*tds.z_cAnion*F_const*(-tds.um_cAnionzr*d(tds.V,r)-tds.um_cAnionzz*d(tds.V,z)),z)-tds.R_cAnion	mol/(m^3*s)	Equation residual	Domains 1–5
tds.Res_cCation	cCationt+d(cCation*tds.z_cCation*F_const*(-tds.um_cCationrr*d(tds.V,r)-tds.um_cCationrz*d(tds.V,z)),r)+if(abs(r)<0.001*h,d(cCation*tds.z_cCation*F_const*(-tds.um_cCationrr*d(tds.V,r)-tds.um_cCationrz*d(tds.V,z)),r),cCation*tds.z_cCation*F_const*(-tds.um_cCationrr*d(t	mol/(m^3*s)	Equation residual	Domains 1–5

Name	Expression	Unit	Description	Selection
	ds.V,r)-tds.um_cCationrz*d(tds.V,z))/r)+d(cCation*tds.z_cCation*F_const*(-tds.um_cCationzr*d(tds.V,r)-tds.um_cCationzz*d(tds.V,z)),z)-tds.R_cCation			
tds.Res_cGO	cGOt+d(cGO*tds.z_cGO*F_const*(-tds.um_cGOrr*d(tds.V,r)-tds.um_cGOrz*d(tds.V,z)),r)+if(abs(r)<0.001*h,d(cGO*tds.z_cGO*F_const*(-tds.um_cGOrr*d(tds.V,r)-tds.um_cGOrz*d(tds.V,z)),r),cGO*tds.z_cGO*F_const*(-tds.um_cGOrr*d(tds.V,r)-tds.um_cGOrz*d(tds.V,z))/r)+d(cGO*tds.z_cGO*F_const*(-tds.um_cGOzr*d(tds.V,r)-tds.um_cGOzz*d(tds.V,z)),z)-tds.R_cGO	mol/(m^3*s)	Equation residual	Domains 1–5

Shape functions

Name	Shape function	Unit	Description	Shape frame	Selection
cOx	Lagrange (Linear)	mol/m^3	Concentration	Material	Domains 1–5
cRed	Lagrange (Linear)	mol/m^3	Concentration	Material	Domains 1–5
cAnion	Lagrange (Linear)	mol/m^3	Concentration	Material	Domains 1–5
cCation	Lagrange (Linear)	mol/m^3	Concentration	Material	Domains 1–5
cGO	Lagrange (Linear)	mol/m^3	Concentration	Material	Domains 1–5

Weak expressions

Weak expression	Integration frame	Selection
2*(-cOxt*test(cOx)+tds.dflux_cOxr*test(cOxr)+tds.dflux_cOxz*test(cOxz))*pi*r	Material	Domains 1–5
2*(-cRedt*test(cRed)+tds.dflux_cRedr*test(cRedr)+tds.dflux_cRedz*test(cRedz))*pi*r	Material	Domains 1–5
2*(-cAniont*test(cAnion)+tds.dflux_cAnionr*test(cAnionr)+tds.dflux_cAnionz*test(cAnionz))*pi*r	Material	Domains 1–5
2*(-cCationt*test(cCation)+tds.dflux_cCationr*test(cCationr)+tds.dflux_cCationz*test(cCationz))*pi*r	Material	Domains 1–5
2*(-cGOt*test(cGO)+tds.dflux_cGOr*test(cGOr)+tds.dflux_cGOz*test(cGOz))*pi*r	Material	Domains 1–5
2*tds.z_cOx*F_const*cOx*((-tds.um_cOxrr*d(tds.V,r)-tds.um_cOxrz*d(tds.V,z))*test(cOxr)+(-tds.um_cOxzr*d(tds.V,r)-tds.um_cOxzz*d(tds.V,z))*test(cOxz))*pi*r	Material	Domains 1–5
2*tds.z_cRed*F_const*cRed*((-tds.um_cRedrr*d(tds.V,r)-tds.um_cRedrz*d(tds.V,z))*test(cRedr)+(-tds.um_cRedzr*d(tds.V,r)-tds.um_cRedzz*d(tds.V,z))*test(cRedz))*pi*r	Material	Domains 1–5
2*tds.z_cAnion*F_const*cAnion*((-tds.um_cAnionrr*d(tds.V,r)-tds.um_cAnionrz*d(tds.V,z))*test(cAnionr)+(-tds.um_cAnionzr*d(tds.V,r)-tds.um_cAnionzz*d(tds.V,z))*test(cAnionz))*pi*r	Material	Domains 1–5
2*tds.z_cCation*F_const*cCation*((-tds.um_cCationrr*d(tds.V,r)-tds.um_cCationrz*d(tds.V,z))*test(cCationr)+(-tds.um_cCationzr*d(tds.V,r)-tds.um_cCationzz*d(tds.V,z))*test(cCationz))*pi*r	Material	Domains 1–5
2*tds.z_cGO*F_const*cGO*((-tds.um_cGOrr*d(tds.V,r)-tds.um_cGOrz*d(tds.V,z))*test(cGOr)+(-tds.um_cGOzr*d(tds.V,r)-tds.um_cGOzz*d(tds.V,z))*test(cGOz))*pi*r	Material	Domains 1–5
2*tds.streamline*(isScalingSystemDomain==0)*pi*r	Material	Domains 1–5

Weak expression	Integration frame	Selection
2*tds.crosswind*(isScalingSystemDomain==0)*pi*r	Material	Domains 1–5

2.4.2 Axial Symmetry

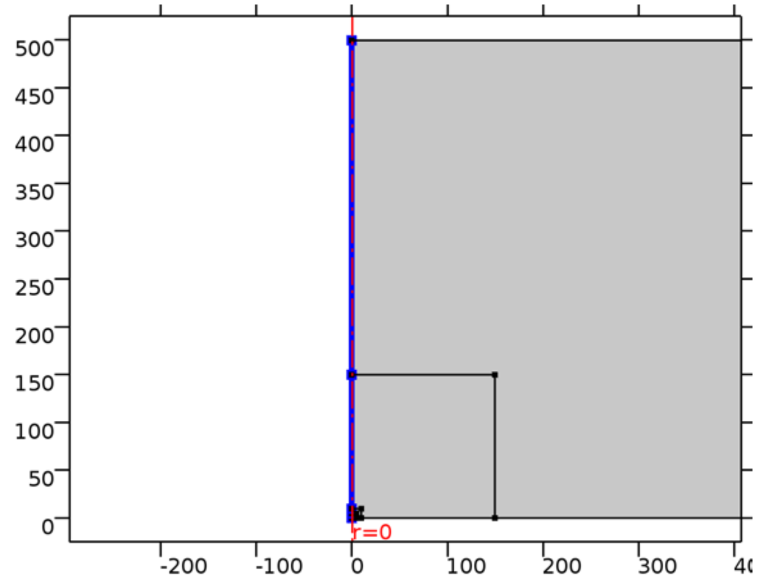

Axial Symmetry

Selection

Geometric entity level	Boundary
Selection	Boundaries 1, 3, 5, 7, 9

2.4.3 No Flux

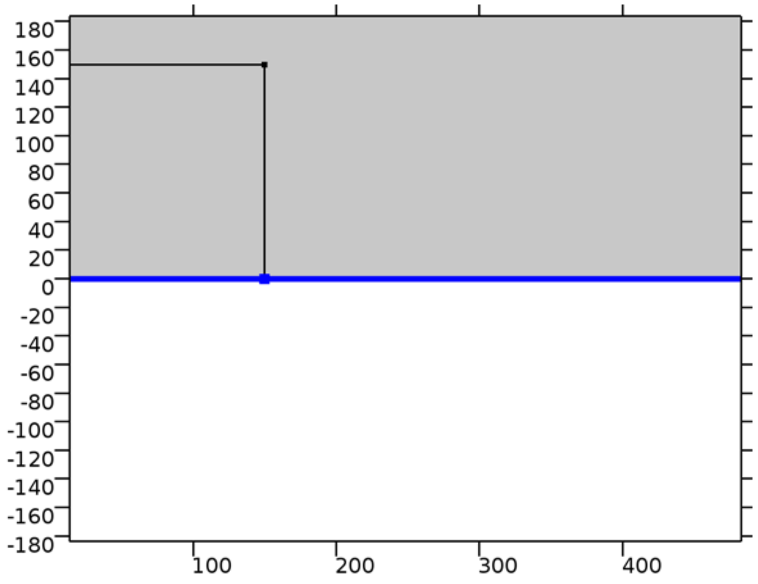

No Flux

Selection

Geometric entity level	Boundary
Selection	Boundaries 13, 16, 18

Equations

$$-\mathbf{n} \cdot \mathbf{N}_i = 0$$

2.4.4 Initial Values

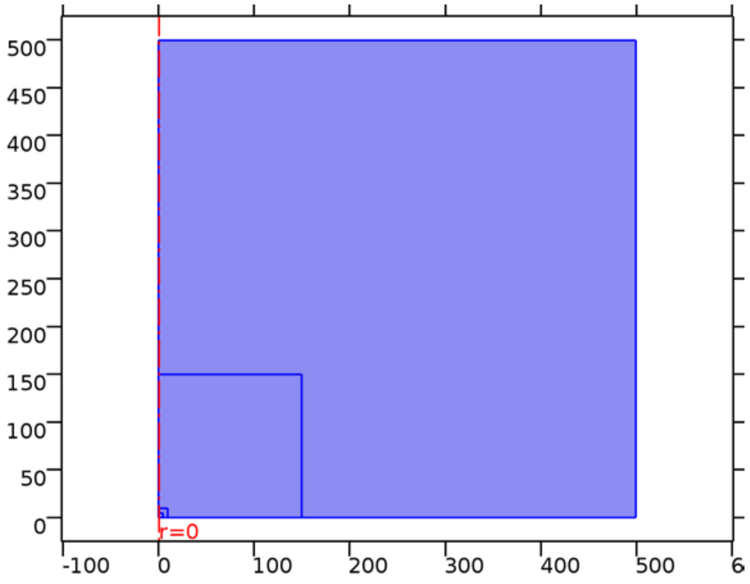

Initial Values

Selection

Geometric entity level	Domain
Selection	Domains 1–5

Settings

Description	Value
Concentration	{0, Cb, Csalt, Csalt, CGO}

2.4.5 Concentration cell boundary

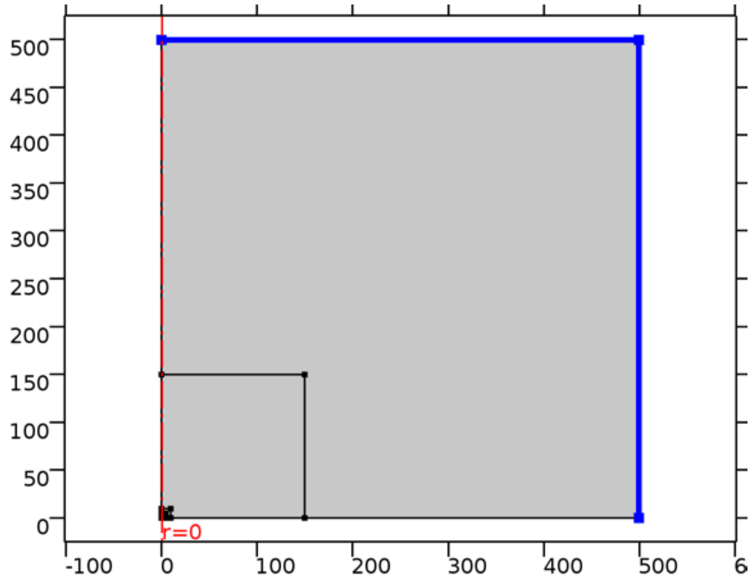

Concentration cell boundary

Selection

Geometric entity level	Boundary
Selection	Boundaries 11, 19

Equations

$$c_i = c_{0,i}$$

Settings

Description	Value
Species cOx	On
Species cRed	On
Species cAnion	On
Species cCation	On
Species cGO	On
Concentration	{0, Cb, Csalt, Csalt, CGO}
Apply reaction terms on	All physics (symmetric)
Use weak constraints	Off
Constraint method	Elemental

Variables

Name	Expression	Unit	Description	Selection
tds.c0_cOx	0	mol/m^3	Concentration	Boundaries 11, 19

Name	Expression	Unit	Description	Selection
tds.c0_cRed	Cb	mol/m^3	Concentration	Boundaries 11, 19
tds.c0_cAnion	Csalt	mol/m^3	Concentration	Boundaries 11, 19
tds.c0_cCation	Csalt	mol/m^3	Concentration	Boundaries 11, 19
tds.c0_cGO	CGO	mol/m^3	Concentration	Boundaries 11, 19

Shape functions

Constraint	Constraint force	Shape function	Selection
-cOx+tds.c0_cOx	test(-cOx+tds.c0_cOx)	Lagrange (Linear)	Boundaries 11, 19
-cRed+tds.c0_cRed	test(-cRed+tds.c0_cRed)	Lagrange (Linear)	Boundaries 11, 19
-cAnion+tds.c0_cAnion	test(-cAnion+tds.c0_cAnion)	Lagrange (Linear)	Boundaries 11, 19
-cCation+tds.c0_cCation	test(-cCation+tds.c0_cCation)	Lagrange (Linear)	Boundaries 11, 19
-cGO+tds.c0_cGO	test(-cGO+tds.c0_cGO)	Lagrange (Linear)	Boundaries 11, 19

2.4.6 Concentration elec boundary

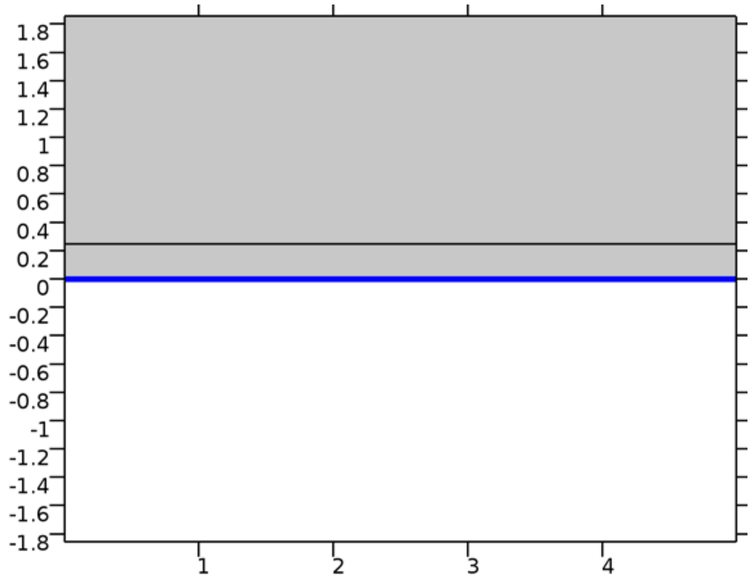

Concentration elec boundary

Selection

Geometric entity level	Boundary
Selection	Boundary 2

Equations

$c_i = c_{0,i}$

Settings

Description	Value
Species cOx	On
Species cRed	On
Species cAnion	Off
Species cCation	Off
Species cGO	On
Concentration	{Cb, 0, 0, 0, 0}
Apply reaction terms on	All physics (symmetric)
Use weak constraints	Off
Constraint method	Elemental

Variables

Name	Expression	Unit	Description	Selection
tds.c0_cOx	Cb	mol/m^3	Concentration	Boundary 2
tds.c0_cRed	0	mol/m^3	Concentration	Boundary 2
tds.c0_cGO	0	mol/m^3	Concentration	Boundary 2

Shape functions

Constraint	Constraint force	Shape function	Selection
-cOx+tds.c0_cOx	test(-cOx+tds.c0_cOx)	Lagrange (Linear)	Boundary 2
-cRed+tds.c0_cRed	test(-cRed+tds.c0_cRed)	Lagrange (Linear)	Boundary 2
0	0	Lagrange (Linear)	Boundary 2
0	0	Lagrange (Linear)	Boundary 2
-cGO+tds.c0_cGO	test(-cGO+tds.c0_cGO)	Lagrange (Linear)	Boundary 2

2.5 Mesh 1

Mesh statistics

Description	Value
Minimum element quality	0.001
Average element quality	0.6579
Triangular elements	15190

Description	Value
Quadrilateral elements	10000
Edge elements	851
Vertex elements	15

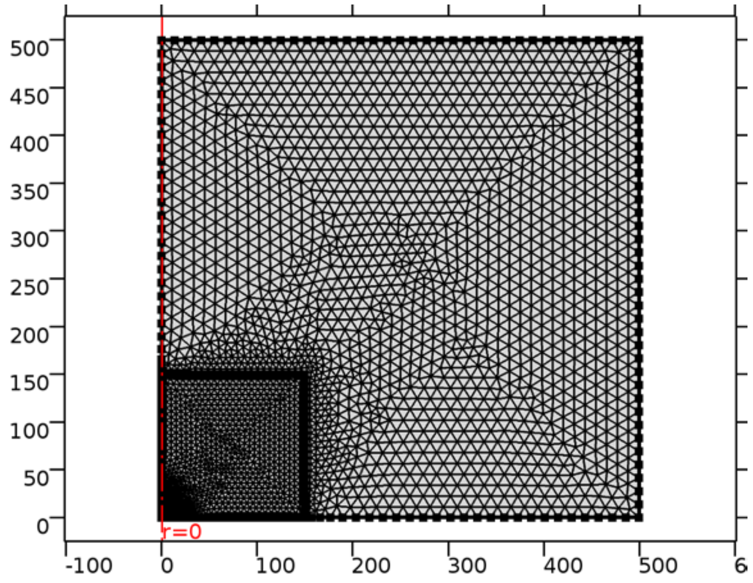

Mesh 1

2.5.1 Size (size)

Settings

Description	Value
Maximum element size	33.5
Minimum element size	0.15
Curvature factor	0.3
Maximum element growth rate	1.3

2.5.2 cell 1 (map1)

Selection

Geometric entity level	Domain
Selection	Domain 1

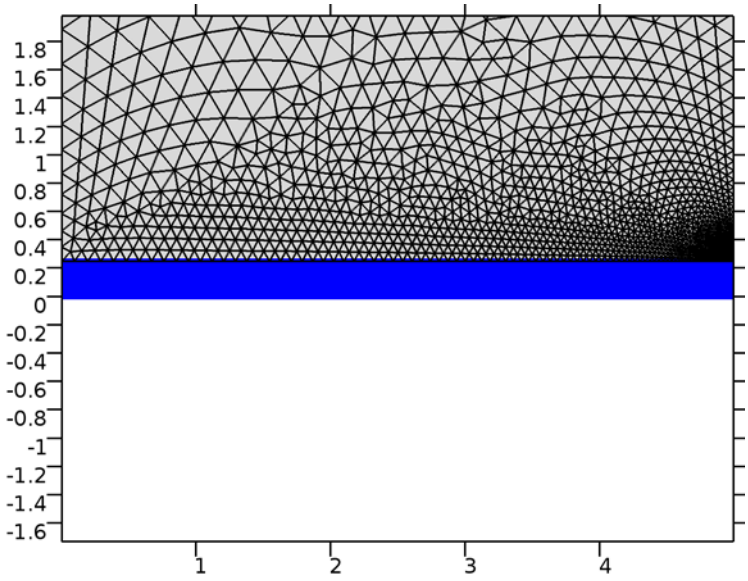

cell 1

Distribution 1 (dis1)

Selection

Geometric entity level	Boundary
Selection	Boundaries 1–2, 4, 12

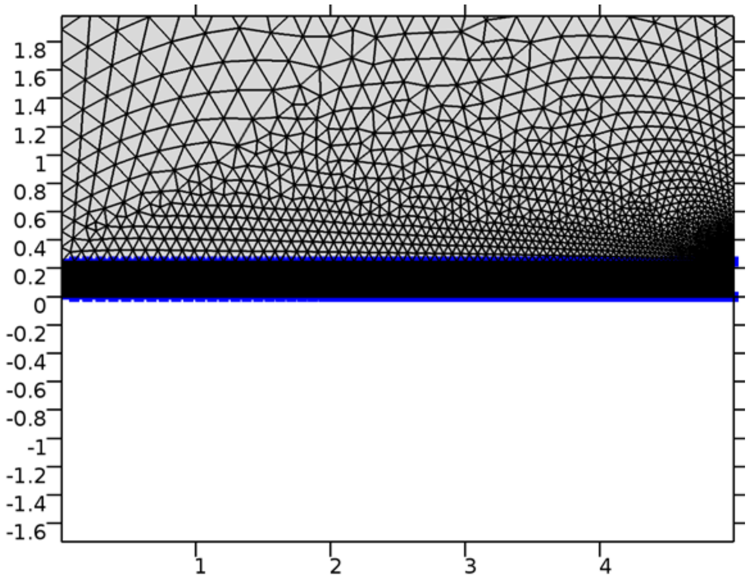

Distribution 1

Settings

Description	Value
Distribution properties	Predefined distribution type
Number of elements	100
Element ratio	100

2.5.3 cell 2 (ftri6)

Selection

Geometric entity level	Domain
Selection	Domain 2

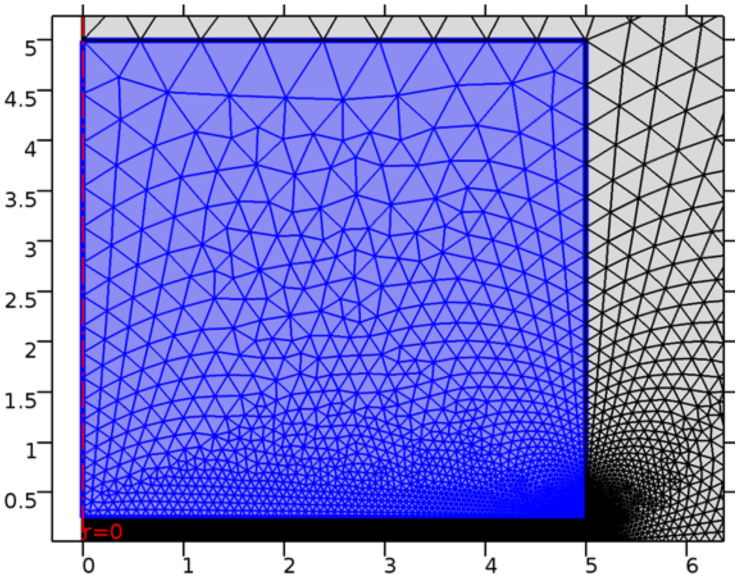

cell 2

Size 1 (size1)

Selection

Geometric entity level	Domain
Selection	Domain 2

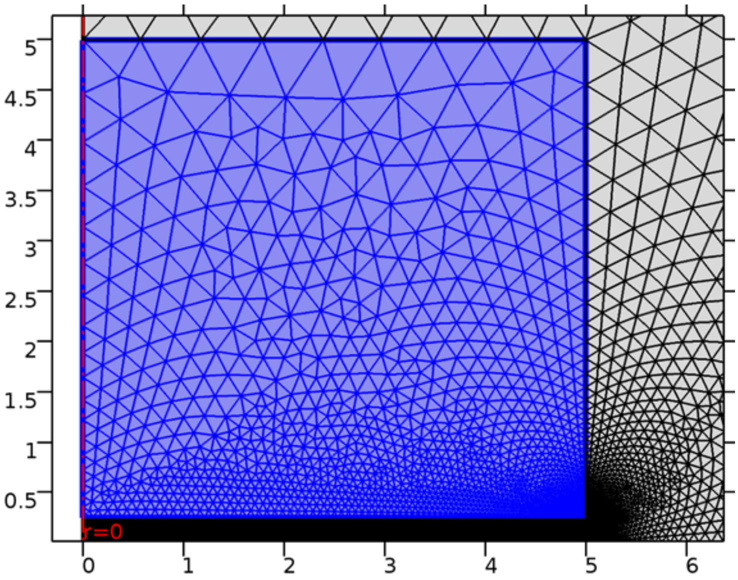

Size 1

Settings

Description	Value
Calibrate for	Semiconductor
Minimum element size	0.2
Curvature factor	0.25
Predefined size	Finer

2.5.4 cell 3 (ftri3)

Selection

Geometric entity level	Domain
Selection	Domain 3

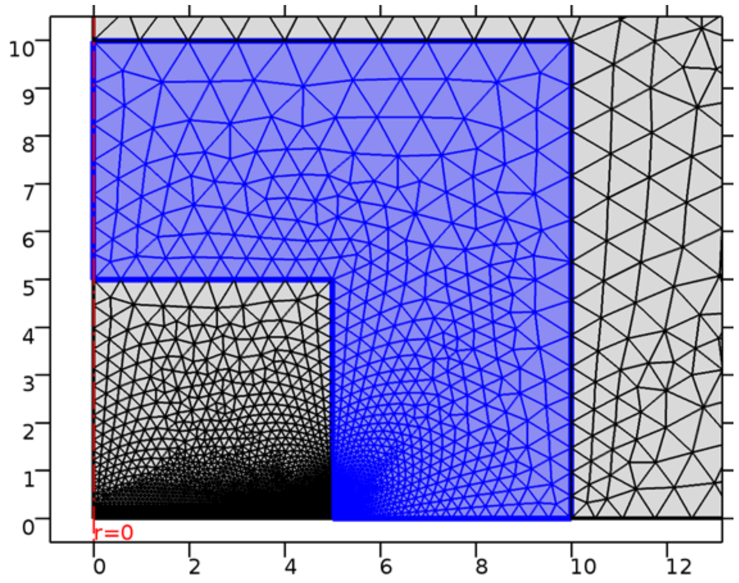

cell 3

Size 1 (size1)

Selection

Geometric entity level	Domain
Selection	Domain 3

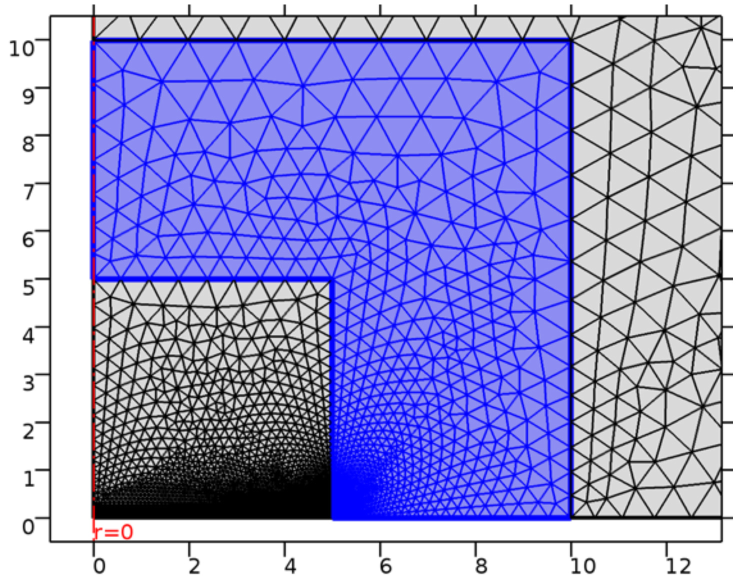

Size 1

Settings

Description	Value
Calibrate for	Semiconductor
Minimum element size	0.2
Curvature factor	0.25
Predefined size	Finer

2.5.5 cell 4 (ftri4)

Selection

Geometric entity level	Domain
Selection	Domain 4

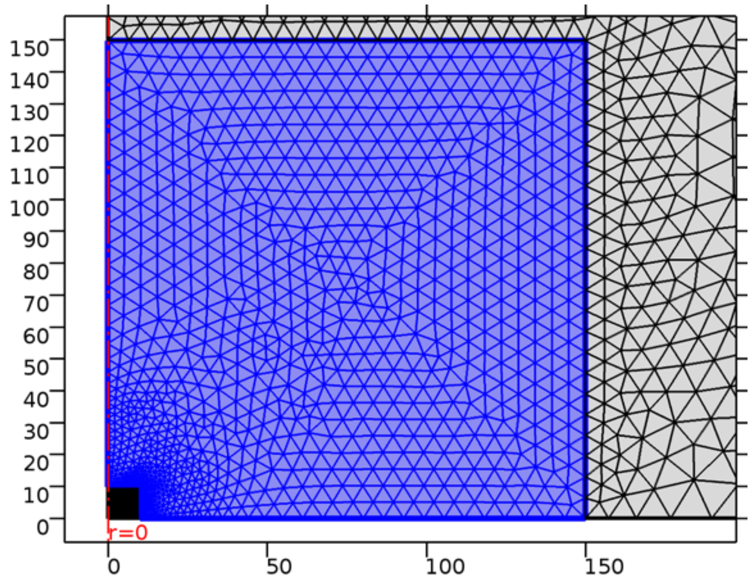

cell 4

Size 1 (size1)

Selection

Geometric entity level	Domain
Selection	Domain 4

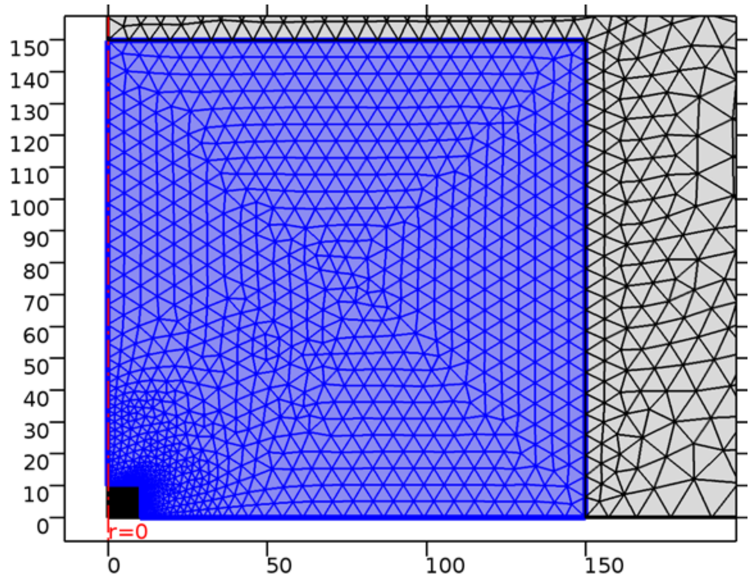

Size 1

Settings

Description	Value
Calibrate for	Semiconductor
Maximum element size	6.5
Minimum element size	1

Description	Value
Curvature factor	0.3
Maximum element growth rate	1.15

2.5.6 cell 5 (ftri7)

Selection

Geometric entity level	Remaining

Size 1 (size1)

Selection

Geometric entity level	Domain
Selection	Geometry geom1

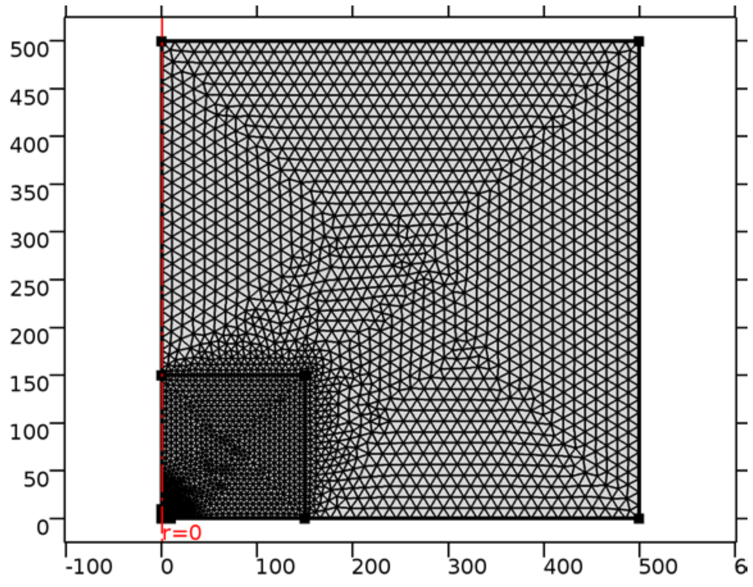

Size 1

Settings

Description	Value
Calibrate for	Semiconductor
Maximum element size	14
Minimum element size	1.5
Curvature factor	0.4
Maximum element growth rate	1.2
Predefined size	Coarse

3 Study 1

Computation information

Computation time	6 h 56 min 53 s
CPU	Intel(R) Core(TM) i7-3930K CPU @ 3.20GHz, 6 cores
Operating system	Windows 7

3.1 Parametric Sweep

Parameter name	Parameter value list	Parameter unit
Csalt	0.05,0.02,0.01,0.005,0.002,0.001,0.0005	mol/L

3.2 Time Dependent

Study settings

Description	Value
Include geometric nonlinearity	Off

Times	Unit
60	s

Physics and variables selection

Physics interface	Discretization
Electrostatics (es)	physics
Transport of Diluted Species (tds)	physics

Mesh selection

Geometry	Mesh
Geometry 1 (geom1)	mesh1

3.3 Solver Configurations

3.3.1 Solution 1

Compile Equations: Time Dependent (st1)

Study and step

Description	Value
Use study	Study 1
Use study step	Time Dependent

Dependent Variables 1 (v1)

General

Description	Value
Defined by study step	Time Dependent

Initial values of variables solved for

Description	Value
Solution	Zero

Values of variables not solved for

Description	Value
Solution	Zero

Concentration (comp1.cRed) (comp1_cRed)

General

Description	Value
Field components	comp1.cRed

Concentration (comp1.cOx) (comp1_cOx)

General

Description	Value
Field components	comp1.cOx

Concentration (comp1.cCation) (comp1_cCation)

General

Description	Value
Field components	comp1.cCation

Concentration (comp1.cAnion) (comp1_cAnion)

General

Description	Value
Field components	comp1.cAnion

Concentration (comp1.cGO) (comp1_cGO)

General

Description	Value
Field components	comp1.cGO

Electric potential (comp1.V) (comp1_V)

General

Description	Value
Field components	comp1.V

Time-Dependent Solver 1 (t1)

General

Description	Value
Defined by study step	Time Dependent
Time	60

Time stepping

Description	Value
Maximum BDF order	2

Fully Coupled 1 (fc1)

General

Description	Value
Linear solver	Direct 1

Method and termination

Description	Value
Damping factor	0.9
Jacobian update	Once per time step
Maximum number of iterations	8

4 Results

4.1 Plot Groups

4.1.1 concentration profiles

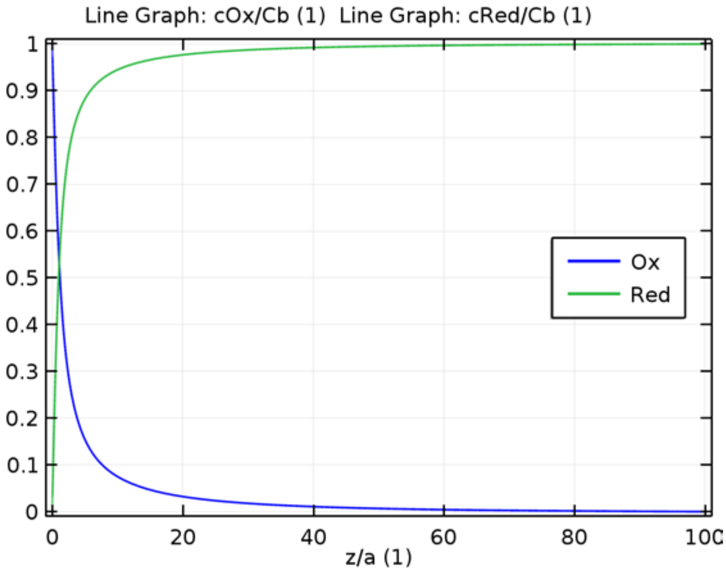

Line Graph: cOx/Cb (1) Line Graph: cRed/Cb (1)

4.1.2 freq & current

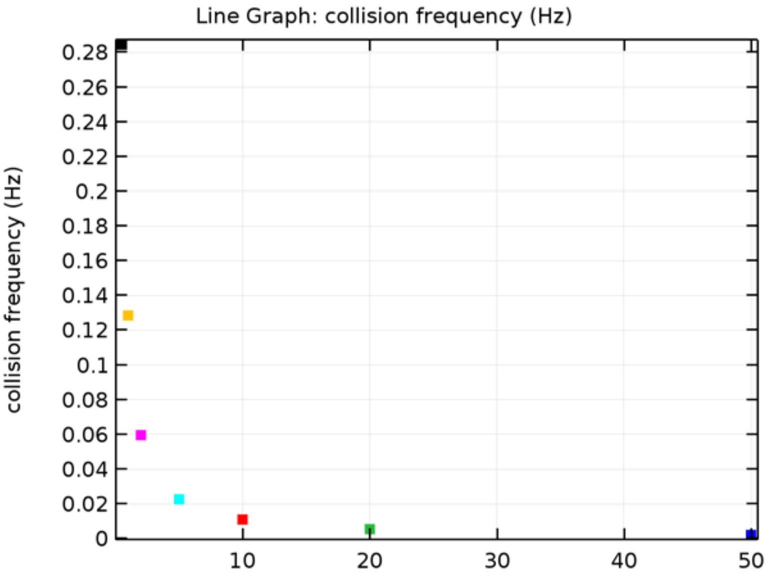

Line Graph: collision frequency (Hz)

4.1.3 potential profile

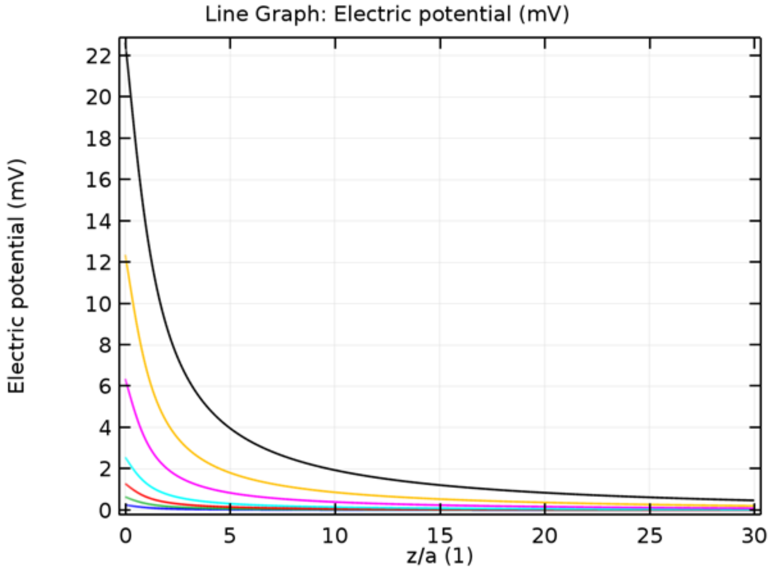

Line Graph: Electric potential (mV)

4.1.4 Concentration (tds)

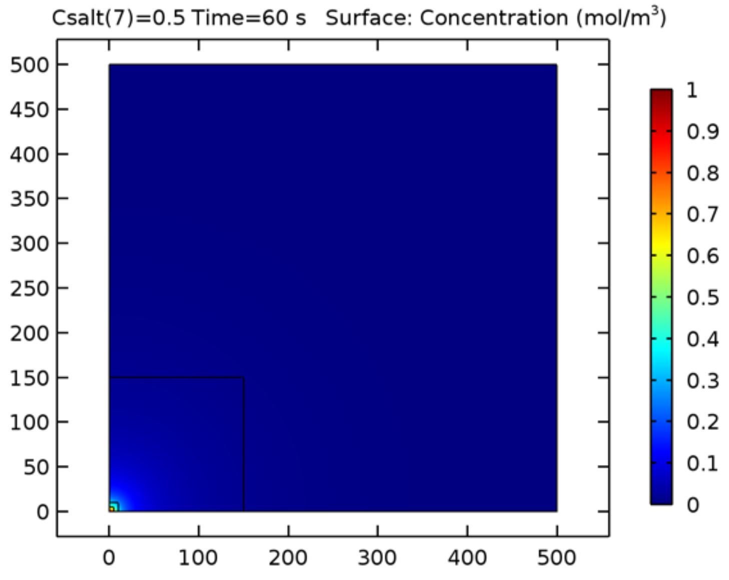

Csalt(7)=0.5 Time=60 s Surface: Concentration (mol/m^3)

2. Step size simulation

Geometry and mesh: The step sizes were simulated in the 3D geometry shown Figure S3. In order to reduce computing time, the size of the cell was reduced by taking advantage of symmetries and using an ellipsoid instead of a sphere or a cube. The size of the ellipsoid was carefully chosen by running a 2D simulation with a large cell (20 times larger than the electrode) beforehand and drawing the iso-concentration surface corresponding to only 1.4 times the size of the electrode. This ratio corresponds to an iso-concentration of 0.52 times the bulk concentration and an ellipsoid with a half-height and half-width of 4.948 and 7.032 µm, respectively. A boundary concentration with 0.52 times the bulk concentration was imposed on the outer face of the ellipsoid. The electrode (blue color Figure S3) is represented by a quarter circle.

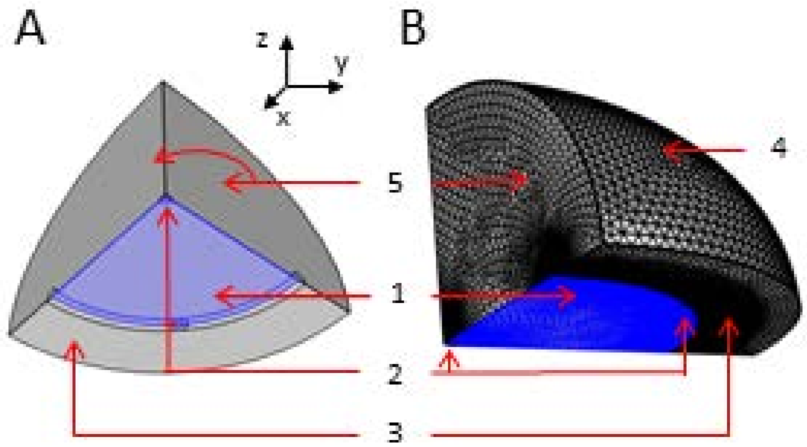

Figure S3. 3D view of the geometry (A) and mesh (B) used for the simulation of the cell and the electrode. The blue boundary corresponds to the electrode. See Table S1 for the attribution of the numbers.

GO sheets were represented as circles, one at the centre of the electrode and the other at the perimeter. In order to increase the quality of the simulation the number element in the mesh was significantly increased near the electrode perimeter and the GO disks where the concentration gradient is high. Typically, the mesh consisted of ≈ 3 million elements. In order to eliminate bias, the same mesh was used to calculate the steady-state current of the bare and blocked electrode.

Boundary conditions and initial parameters:

Table S1. Parameters of the simulation

Boundary	N° in Figure S2	Value
**cell**	4-5	[FcMeOH] = 0.52 mM
**electrode**	1	[FcMeOH] = 0 mM
**glass sheath**	3	dc/dz = 0
**GO sheet**	2	dc/dz = 0
**Initial concentration in the cell**		[FcMeOH] = 0.52 mM

Equation solved and step calculation:

To calculate the concentration of FcMeOH at the surface of the electrode, the stationary form of Fick's second law was solved:

$$\nabla \cdot (D \nabla \mathrm{c}) = 0$$

Here *D* and *c* are the diffusion coefficient (6.7 x 10^{-6} cm^2/s) and the concentration of ferrocene methanol, respectively.

Step size calculation: The step size was measured as follows. First, the steady-state current of the bare UME was simulated. The current was calculated by integration of the FcMeOH flux over the surface of the electrode and then multiplying by 4**D***F,* where *F* is the Faraday constant and the factor 4 accounts for the four quadrants of the disk-shaped UME.

$$i_{ss}^{bare} = 4\, i_{1/4}^{bare} = 4FD \iint^{electrode\ surface} \left(\frac{dc}{dz}\right)_{z=0} dxdy$$

Then, one of the GO sheets was set to no flux boundary and the current was again calculated. The two GO sheets were alternatively set to no-flux boundary conditions to calculate either the current step at the centre or at the edge of the electrode. When the sheet was positioned at the centre of the UME, the total current was simply four times the current measured on one quadrant. However, for the sheet positioned on the edge, the total current is given by:

$$i_{ss}^{edge} = i_{1/4}^{edge} + 3\, i_{1/4}^{bare}$$

where $i_{1/4}^{edge}$ is the current with a sheet at the edge. The step size is finally defined as:

$$\Delta i_{step}(\%) = 100\, \frac{\left(i_{ss}^{edge/center} - i_{ss}^{bare}\right)}{i_{ss}^{bare}}$$

3. 2D vs 3D blocking

<u>Geometry and mesh:</u> A 2D axial geometry was used to represent the UME and the solution above. Following the same strategy as used for the step size calculation, the volume of the cell was described with an ellipsoid of half-width and half-height of 2.17216 and 1.93441 µm, respectively. The iso-concentration at the surface of the ellipsoid was set to 0.7 mM, corresponding to a concentration of 1 mM at infinity. The 3D and 2D blocking objects were represented by a circle and a line, respectively. In cylindrically symmetric geometry, the circle and the line correspond to a toroid and a ring, respectively. The mesh was refined near the edge of the electrode and the blocking object. A mesh with 2.3 million elements was used.

<u>Boundary conditions and initial parameters:</u>

Table S2. Parameters of the simulation

Boundary	Value
**cell**	[FcMeOH] = 0.7 mM
**electrode**	[FcMeOH] = 0 mM
**glass sheath**	dc/dz = 0
**GO sheet**	dc/dz = 0
**Initial concentration in the cell**	[FcMeOH] = 0.7 mM

Equation solved and flux calculation:

To calculate the concentration at the surface of the electrode the stationary form of Fick's second law was solved:

$$\nabla.(D\nabla \mathrm{c}) = 0$$

The flux of FcMeOH at the electrode surface is given by Fick's first law:

$$flux\ = D\left(\frac{\partial \mathrm{c}}{\partial \mathrm{z}}\right)_{z=0}$$
